# Supplementary material for: Beyond silos: Drivers and barriers to intersectoral collaboration in zoonotic disease surveillance and response in the Greater Accra Metropolitan Area, Ghana
Source: PLoS One. 2026 Apr 16;21(4):e0347471. doi: 10.1371/journal.pone.0347471 (PMC13086352; doi:10.1371/journal.pone.0347471)
Supplement: S1 Appendix — (PDF) [file pone.0347471.s001.pdf]

S1 Appendix

Interview Guide for *"Beyond Silos: Drivers and Barriers to Intersectoral Collaboration in Zoonotic Disease Surveillance and Response in the Greater Accra Metropolitan Area, Ghana"*

This interview guide was administered to purposively sampled actors from the human health, animal health and wildlife health sectors, all of whom work directly at various levels of zoonotic disease surveillance and response in the Greater Accra Metropolitan Area, Ghana.

Part 1: Introduction

1. **Introduction to the research** – Explain the purpose of the study and introduce the research team.
2. **Participant information and clarifications** – Allow participants to read the participant information sheet and ask questions.
3. **Consent process** - Obtain permission from participants for participation, audio recording, and if required, photographs.
4. **Signing consent forms** - Participants formally agree to participate.
5. **Demographic data collection** - Participants fill out a demographic data form.

Part 2: Intersectoral Collaboration in zoonotic disease surveillance and response (ZDSR)

Collaboration scope and actors

1. **Do you collaborate with other sectors in ZDSR activities?**
   - If yes, with whom and how do these collaborations typically occur?
   - *Probe for explicit descriptions and details.*
   - *Probes: human health, animal health, environment, wildlife, media, education, security services, NGOs, the assembly, etc. Include any other new actor/sector mentioned in other interviews*
   - *Note down specific examples for follow-up questions.*

Collaboration Activities and Processes

2. **In what specific activities related to ZDSR do you collaborate with other sectors?**
   - *For each area, ensure that participants are only referring to* ***district/subdistrict*** *level collaborations as some participants may work at other levels and may conflate the activities.*
   - *Use the following as prompts to trigger participants' memories. Prioritize the most relevant ones to avoid participant fatigue.*
     - ***Coordination*** *- e.g., of persons, units, departments, agencies, committees of an outbreak response involving other sectors.*
     - ***Surveillance technical functions*** *– e.g., joint activities related to outbreak detection, response, control*
     - ***Scientific support*** *– e.g., seeking technical advice from another sector, conducting research together, etc.*
     - ***Protocol design*** *– e.g., joint activities related to the designing SOPs, job aids, etc.*
     - ***Sampling activities*** *- e.g., joint activities related to the taking, packaging or transporting of human/animal samples*
     - ***Laboratory activities*** *- e.g., joint activities involving any laboratory of another sector (case confirmations)*

- ***Data analysis and interpretation*** *– joint activities related to the analysis and/or interpretation of another sector's data*
- ***Data management and storage*** *– joint activities related to the storage and/or management of surveillance data*
- ***Exchange/Reporting of surveillance data (routine)*** *– activities involving sharing or receiving surveillance data to or from another sector. Take note of the frequency.*
- ***Communication*** *- e.g., modes, frequency*
- ***Dissemination*** *- e.g., events, joint visits to media platforms to share zoonoses, collaborative visits to schools, etc.*
- ***Other collaborative areas*** *- e.g., trainings, meetings, planning, resource sharing etc.*

Collaboration Beyond Disease Outbreaks

3. **Have you collaborated outside of outbreak situations?**
    - If yes, in what contexts and for what purpose

Collaboration by Disease Type

4. **Have you had a ……. case before?......... If yes, did you collaborate with any sector during that time?**
    - *Prompts: Rabies/Dog bite, Anthrax, Avian Influenza, Zoonotic Tuberculosis, Lassa fever, Ebola Virus Disease, Yellow fever, Dengue Fever, Trypanosomiasis.*
    - Are there any **other** diseases that I have not mentioned yet where you worked with another sector?

Motivations and Goals of Collaboration

5. **What motivates you to collaborate?**

    What drives you to work with others?

6. **What do you aim to achieve through collaboration?**
    - *Prompts: improving performance, effectiveness, etc.*

Expected Benefits of Collaboration

7. **What benefits do you expect from collaboration?**

- If already collaborating: what do you believe collaborations bring to your work?

Barriers to collaboration

8. *If participants mention that they do not collaborate*, ask: **Why do you not collaborate with [specific actor]?**
    - If applicable, ask: Have there been situations where you collaborations could have happened but did not? If yes, why?

   **Captures missed collaboration opportunities and deliberate non-collaboration*

Positive Collaboration Experiences/Outcomes

9. **Can you share specific examples of successful collaborations**
    - What were the positive outcomes of these collaborations?

Final Thoughts

10. **Is there any other thing you would like to share?**
    **Encourage participants to reach out if they remember any important information post-interview.*

THANK YOU VERY MUCH FOR YOUR TIME!
