# Supplementary material for: Beyond silos: Drivers and barriers to intersectoral collaboration in zoonotic disease surveillance and response in the Greater Accra Metropolitan Area, Ghana
Source: PLoS One. 2026 Apr 16;21(4):e0347471. doi: 10.1371/journal.pone.0347471 (PMC13086352; doi:10.1371/journal.pone.0347471)
Supplement: S1 Table — (PDF) [file pone.0347471.s002.pdf]

S1 Table - Supporting quotes for identified factors influencing intersectoral collaboration in zoonotic disease surveillance and response in the Greater Accra Metropolitan Area of Ghana.

#	Factors	Sub –Themes (Codes)	Supporting Quotes
1	**Interpersonal Relationships**	Good (personal) rapport	• *"It's because we have the rapport..." (AH)*<br>• *"I'm in good terms with the director and other staff" (AH).*<br>• *"Because of [name], but when he died, I stopped it [routine data sharing and communication]" (AH).*<br>• *"… but the truth is Dr. [name of AH] and I are very good friends. There's a personal touch"[…]he and I … are the newest […] in the assembly, we are kind of the newest addition … Yeah, we work hand in hand, you know,[in] everything; What do you think? Let's do this, let's go this way … we work hand in hand" (HH).*
		Informal networks and personal incentives	• *"The first man didn't help but the second [district assembly] coordinator, had a [dog]. So, when he came [to] a meeting he said 'ah, where is the vet?' and I said 'me', so I treated his animals for him and because he liked animals, whenever it came to vaccination he was there to help. The trick we used was that, most of the coordinators who came, they had dogs and animals, so we made sure that we treated their animals for them. So, whenever we put in our application, it becomes easier." (AH)*<br>• *"With the bird flu […] apart from the health people, we inform NADMO, and sometimes[the] police […] if the person wants to prove difficult […]. The commander is a friend, he has a dog. So I take care of it. And in order for me to get his yes [approval/support], I know how to deal with him. So when I need the help then he [will] also quickly mobilize his people to help us." (AH)*
2	**Professional Hierarchy and Status Perceptions**	Negative perceptions of professional superiority	• *"The medics feel they're tin-gods" (AH).*<br>• *"You know in our country here, the medics always rate themselves higher" (AH)*<br>• *"Right from the university, you know at Tech [Kwame Nkrumah University of Science and Technology] they will tell you that the Agric students; we are dissappointed medical doctors" (AH)*<br>• *".. I feel the health, the environmental and all, I feel they're really working together, but then it looks like …, they side-line us or it's like they don't see the importance of the vets. That's what I feel personally"(AH).*<br>• *"it's like they don't see our importance or so, it makes things difficult. It's like you don't even have a voice. You're in a meeting and you can't even have a voice. […] that's how I feel. […] A lot of the meetings, you go and sit in, and it's like you're not even there. I mean initially, 'ohh yeah the vet is here, everyone; oh nice nice' that's it. It's like your job is not as important as the other sectors" (AH)*
3	**Financial Resources**	Expectations of remuneration	• *"I used to go there to call them [HH] to accompany [me] so that they would do screening. But nowadays… when you go with them …you must give them something. In fact, my boss was doing that a lot, so when my boss left, I also did some—I think about two times—then I told them, 'Me, I'm a small boy, I don't have money to be giving to them.' […] Once they are*

		for surveillance activities	going out of station, they may want some allowance. Then I told them, [for] myself, when a case is reported and I'm going, I use my own money for fuel, and nobody gives me anything. Uh-huh, so it's not possible we can continue with that." (AH)<br>• "… since then [past meeting with HH] they have not called me. Even [for] that one, they promised to give me an allowance, it didn't come, so maybe that's why they are not calling me anymore." (AH)
		Resource disparities between sectors	• "This is the problem. They don't have a place to even keep a dog for observation, they don't have any place. [So, if you have a dog bite case, you let them know?] We let them know but they are not going to do anything about [it]" (HH)<br>• "Certain times, you'll call them to collaborate, they need basic things that will help them. Some of them, when you're involving them, they need the logistics before they can go, but this is the case [where] we [also] don't have." (HH)
		Perceived profit-driven collaboration	• "Animal health, yes, environmental health, no. [Why?] There's no money in it for you [them]. They only come in when there is money […] If it's not something that will directly benefit them, or there's money coming to them out of it, they're not bothered." (HH)<br>• "If you talk to them, they won't do anything. They'll tell you 'Oh, director, let's go and do some advocacy, lets write a memo and send it to the assembly so they sponsor us to go and do some advocacy…'. [Do you write the memo?] Me? No. Because if I should [do] that, they'll go and take the money and you'll not get the money to do the work. So I don't do it" (HH).<br>• "Even when the money comes, they want to take your money". (AH)
**4**	**Surveillance Governance and Organisation**	Surveillance organization issues	• "See, in reality, the systems must be in place because you see we have systems for reporting. But if my system of reporting, doesn't include somebody there's no way I'll go and root that person [out]" (HH).<br>• "It [collaborations]is usually propelled by something, it's propelled by something" (HH).<br>• "But you see, there has to be something to cause us to do that communication" (HH).
		Lack of political will and institutional support	• "Yea they don't seem to have the political will. I don't know if they don't understand the implication of certain things but sometimes you expect them to stand in and do things, they don't do it. This rabies thing for instance, Mr. [name of AH] and myself, we met them, we spoke about culling some of the dogs because we have a huge stray dog problem in this district. We talked and talked and talked and talked, they asked him to come up with a plan, he did it, nothing happened. The assembly didn't make any effort." (HH)<br>• "I pushed and pushed up to a point and then I left it alone, because I realized that in the end [it would look] like there's something in it for me because somebody said maybe I was expecting some benefits from it meanwhile I don't even live in this district. So I was like, I mean, if you who live here are not seeing the gravity of[the] problem. I mean, I just left it alone." (HH).
		Lack of continuity in surveillance efforts	• "so this [intersectoral] committee was formed just last year, it should have been in existence long ago, but it looks like the old one that was in existence - when the people retired or were transferred out, it was never reconstituted" (HH).
**5**	**Workforce availability**	Workforce Scarcity	• "You have to ask yourself; how many veterinary officers are there? [One] How many environment officers are there? [A lot] Exactly… So in as much as we may have a veterinary officer, you have to find out how useful [it is] … because it's

			probably just one person, how much can they do? But as for environmental officers, they are way around [they are plenty].” (HH).<br>• *“I think they [animal health sector] are not much you know … I think it's the capacity… Because I think one person is not enough to do the job.” (HH)*<br>• *“… and animal [health] people seem to be out in most districts.” (HH)*<br>• *“I can even say that I collaborate sometimes with the other districts… because they don't have a veterinary doctor there but they have the technicians. So, the technicians sometime call me if there's a case around.” (AH)*<br>• *“I’m the doctor without technicians … I don’t have a tech” (AH).*<br>• *“… maybe because the district got separated, and maybe because we didn’t have a full complement of the veterinary staff, that is how come. Even the Mr [name of AH] that I knew, now I don't see [him]. So when the district got separated, I think officers had the choice to choose where they want to be, and maybe, unfortunately the new district didn’t get a veterinary officer.” (HH)*
**6**	**Personal Initiative and Assertiveness**	Assertiveness as a strategy to foster collaboration	• *“Because right now I have environment here[on the phone]. She's trying to reach me […] she needs some information on something, she'll ask me. I need something, I will ask her […]. So that effort is made from both sides, so we are able to share and then move on together. It isn't the same with animal [health],[…]they probably feel they don’t really need us, you understand?” (HH)*<br>• *“I'm vocal. I went there during the COVID time [and] introduced myself. I was introduced to the whole team […]. So that rapport was set up- was already there. Wherever I am, whether you like it or not you will like me because I'm inquisitive and that is how scientists should be. You should investigate, you shouldn't coil back and think they will call you; they won’t call. That’s the situation we have in Ghana.” (AH).*<br>• *“what I did was that, when I went [started work], I went [and] introduced myself to the health people” (AH).*
		Perceived Responsibility Shift as a barrier to collaboration	• *“What are the animal health people too doing to collaborate with us? They should also contact us” (HH).*<br>• *“When there’s an outbreak [where] maybe we’ve had a case … [like] lassa fever, we expect that the vet, animal health, wildlife or environment will rather come in and work with us. [Okay, so you expect them to make the move?] Yes” (HH)*
**7**	**Familiarity and Institutional Visibility**	Accessibility and Visibility as facilitators	• *“We collaborate more with the environmental health because they are the ones we meet, the ones we know.” (HH)*<br>• *“When I came here, I was sent to everybody, we introduced ourselves so they know me and then the lady in charge of public health [also knows me] […]. So now because of public health, anything that they are doing, she would just give me a heads up. Then she formed me that I'm a member of the Rapid Response Management team. So, we formed a WhatsApp group, any meeting they put it there, she shares invitation and when we attend, we discuss issues. We were there when COVID came, when Ebola was trying to shoot its head, we met again to discuss the rapid way of solving it should in case it comes” (AH)*<br>• *“The medics at [name] and other places, because we have met in some programmes and […] because of exposure with some of us […], I remember […] the doctor in [name], […]so we had a meeting […] and I talked about rabies. Since then, when there were cases, he used to bring it up, because he knew now.” (AH)*

			• *"Ah it was one of these assembly meetings. I think [a] unit head meeting […]. […] we were discussing budgets and then I heard that they had used some money to vaccinate dogs and then I asked them the number of dogs they vaccinated. And I juxtaposed that because I said, 'look, this is the number of dog bites we've had. We definitely have a lot of dogs. So how is it that you vaccinated [this number]?' So, it's not like I even routinely knew; it was just because we were discussing budgets[…]. [So because of] that one meeting … I came and looked at the dog bites [data] later and then I called him that, 'let's talk, let's talk', yeah. [So]I invited him here, the Vet […]. The trigger was the budget for vaccination" (HH)*<br>• *"You see why the environmental officers come in all the time? Because […]they are the people we get to all the time, so that they also link us with […] other people, ahaa so that's how come. They are always everywhere" (HH)*<br>• *"There's easy access to them…We know where to find them[…],by phone and physical as well" (HH).*<br>• *"... yeah, so you'll find environment … you always find them" (HH).*
		Unfamiliarity and limited visibility as barriers	• *"Personally, I don't have her [AH] contact, maybe she is on it [joint district Whatsap surveillance platform] or maybe she's not on" (HH)*<br>• *"so they too where are they? […] Who else is the focal person? Because any of the other things, when you take ambulance, we know who the focal person is, NCCE [National Commission for Civic Education], we know who to reach, environment, we know who to reach. Why is it that [for] the wildlife, we don't know who to reach? So, it means that from their end too something is missing" (HH)*<br>• *"To be honest, I'm not sure I've ever seen the vet in the district" (HH).*<br>• *"Me I have never met her [AH]. And so far since I've been here, we've had two epidemic Management Committee [meetings] during the […] outbreak. We had ... a meeting and then during the COVID also but during these two meetings I have never met her" (HH).*<br>• *"You know animal [health] sector where are they? Where have they been hiding?" (HH)*<br>• *"But when it comes to response, normally veterinary services are missing … they are normally not visible" (HH).*<br>• *"[for] dog bite cases, that's why I was saying that because we don't have the office, the collaboration wasn't there" (AH)*<br>• *"If we have an office, you know; it's an office you can come to. ... Yes, we try so hard but it's like, I mean, imagine ... every day you go and sit under a tree, ... and there's nothing, like nothing; you're just there, nothing, not at least an office... people coming to the office, ... for help, it will make it much easier and like our presence will be known. But just like there, like go to the field, that's it" (AH).*<br>• *"Can you imagine our clinic has been destroyed to construct stores […] they destroyed it to build stores." (AH)*
**8**	**Public Health and One Health Knowledge and Training Systems**	Public Health training as a catalyst for collaboration	• *"I think those… involved [in the rabies case] were people who have had Public health training. The person who was managing the case,… were people who had had this GFELT [Ghana Field Epidemiology and Laboratory Training] program [along with] their colleague vets so they called. So those who were trained, who knew about rabies because of the GFELT program[…], or their knowledge in public health,[…] were able to call their colleagues in vet. Probably during the course of the training [the] vet talked about rabies and now that's why [they engaged AH in managing the case]." (AH)*<br>• *"Yes, but that one [past communication with HH] was not even frequent. But I think for some few [it is]because she also did public health. So I think because of that". (AH)*

			• *"I remember when I went to the hospital first, the polyclinic, and I started educating the doctors, and […] then they came to realize that, this thing that we are doing, we should always involve the vets" (AH).*<br>• *"[…] so they won't call you. But you have to educate them on what to do. And believe you me, after I took them through somebody coming with first rabies, dog bites, what they should do, they were recording like how you're recording"(AH).*
		Personal initiative in knowledge acquisition	• *"This One health thing, you know, I learnt of this one health thing through my [own] learning … I heard it once, I mean, it's really established in one district in Greater Accra, but the other districts, me I heard it and I read it up, I read about it. But to say that we've had training on it, no" (HH)*
		Perceived impact of public health knowledge on willingness to collaborate	• *"I've noticed that the medics, those who have done public health, their attitude is different from those who have not done public health. […] so those I was having problems with [are] those who have not done public health, so after doing public health for two years, when they come to me, they respect me" (AH)*<br>• *"But it will interest you to know some health professionals don't even understand what veterinary does and what we do… Some people might ask you "ah so this one too you go to school to become a veterinary officer?" I went to [hospital name], my wife had a caesarean section, so I was saying that we also do caesarean section and some of the nurses, … were surprised… I told them we do spaying and serotonin and those things then one doctor was passing [by], "doc come and listen o, they said they also do this thing'… So I think even the health professionals themselves, a lot of them … also need sensitization and we the vets too, so that there can be that collaboration" (AH).*
		Sectoral silos & the overlooked role of other sectors	• *"Wildlife? They are even out of the picture …People have pet monkeys but to think of working with wildlife, I'm telling you the truth, it hasn't even occurred [to me] …. I do [collaborate] with all, except wildlife but you've given me a new perspective" (HH)*
**9**	**Shared Interests and Alignment.**	Shared interests as a driver of collaboration	• *"[…] We are new, our perception is different from others. My views are almost the same like hers, she went to the field this morning and when they came back […], she wanted to brief me on what happened, so I made my comments. And it is actually good, because at the end of the day, its's going to help all of us." (AH)*<br>• *"The MCE is very much interested in … public health. I know so, because …anything health, our recent annual review - he stopped everything he was doing and came to sit through and he was taking notes and making remarks and making suggestions [on]ways to resolve some of the issues that came [up]. He was very interested in our data; how do we capture and present data. And in a peer reviewed thing [session] that we did, he was so blown away. So we found that this [is] someone who is interested and how he holds the various departments under him to also be accountable." (HH)*
		Lack of common interests as a barrier	• *"Because we get our own data, based on what we get from the hospital so we don't […] really feel like we need it [animal health sector data]. (HH)*<br>• *"[…] I work with them on a as it is required basis. When we were doing the District Health Committee, I made sure they were part of it mainly because I felt that it is very important that anything we do, we work with environmental health.*

			However, the issues raised there, nothing. They won't do it; it doesn't concern them. They will say yes at meetings to decisions but they won't do it." (HH)
**10**	**Individual Motivations**	Practical benefits of collaboration	• *"I hear my colleagues complaining about the Agric staff and the director, and I say no, if you work in tandem with them, you will get benefits, it will help you because it's not everywhere you can reach but they are many so if you are in good terms with them, there are a lot of things that you can get. So that's what I do, so me, I'm very nice with Agric director and then the rest of the staff" (AH)*<br>• *"[...] because they are more than us and there are some places that they reach, we cannot reach. So with the collaborations,[..] they will come and call me, [that] there's a problem here. So I need them and it's working very well for me, yes" (AH)*<br>• *"Because they [environmental health] are the people that […] we get to all the time, so that they also link us with […] other people" (HH)*<br>• *"we have even gone to the vet office to go and look at data. [...] We've heard the information [report of a case], […] ours is the health so [we wait] until that person comes to the health and report[s] that I have been bitten by a dog […] then we take it up. They[AH], don't get it[the alert or information]. So we thought okay -that is just half of the data, but when you contact the vets, you would be able to get the full detail. Yes, [the] full detail of what is happening in your districts." (HH)*<br>• *"And sometimes when we need data too we fall on them [Agric department], because we alone cannot reach everywhere." (AH)*
		Personal & Institutional Recognition	• *"And they also get excited when they hear that the Vet man has gone to [do] this and [to]that school to go and do education and when they hear that, they're excited. Because at the end of the day, it boosts their image also up"(AH)*
		Obligation and compliance	• *"I used to work at [hospital], the clinical sector and even then I remember we were told that when we have suspected [should] contact [ ], the Metro vet" (HH)*<br>• *"With me, with my background and training back in school, we were already trained on this surveillance that if there's an outbreak, you need to involve the relevant stakeholders" (HH)*<br>• *"so if, if it's a suspicious dog bite, we would call them [AH] in. And our expectation was that they would either give us the vaccination history of the dog or keep the dog, test the dog or something. They don't have facilities for any of those. ...[So, at least you call?]...We will call just to cover my back" (HH)*<br>• *"Because on your destruction form, you need them [NADMO] to sign a document because without the form signed by the NADMO director, you can't go and destroy [the birds] and you can't compensate [the farmers]. I did one[destruction and] I didn't inform the NADMO guy, ask him how we suffered. So now I had to go to the NADMO director and explain. He said he knew so he was just looking at me." (AH)*<br>• *" when they [HH] go and meet a stumbling block then they call you that this is what has happened, so what should we do next?" (AH)*

11	**Positive Collaborative Outcomes**	Improved response and control measures	• *"When we inform, I remember [a recent dog bite case]. So I think the district, I don't know whether it was [the] assembly or somebody, but they were able to purchase vaccines and then immunized some of the dogs (HH)*<br>• *Okay, so because of the outcome of our research, so when the cases kept on coming in, we also went to the community, [and] did a lot of assessments. Then this thing came out that we should vaccinate the dogs, especially in the area where the bite came from. So they organized the NCCE, a lot of announcements was made in the area. And then the vaccinations took place….. (HH)*
		Enhanced surveillance and early detection	• *"So with the collaborations, sometimes if I need data or I need something they will come and call me, they will call me [that] there's a problem here.... (AH)*<br>• *I remember when the AI came like this. We didn't know where they got to know. I think that we, there was a farm we had not even confirmed in our place, but rumours... So there have been a lot of cases where, because of [our] collaboration, we've been prompted. Sometimes, I remember last week like this, the cat case that we had, it was the health people that called me... [So you would not have even heard of the case?] No. (AH)*
		Improved access to vaccinations	• *"… Veterinary has been helpful as far as dog bite and rabies is concerned. Once we send someone there, they give the vaccine [for] free. If the dog is rabid ... They give it free from there." (HH)*<br>• *"…But when they go to the veterinary office, they do the lab on the dog to see ... Once this is positive, or there's a possibility of it being rabid, they give the vaccine for free". (HH)*
12	**Stakeholder Aspirations for future collaboration**	Greater efficiency and effectiveness	• *"Oh, but at the end of the day it will make our work smooth...and easier (HH)*<br>• *"I think it will make the work easier."(AH)*<br>• *"We are working as a team.[...]. These people [other sectors] have a lot of knowledge with different backgrounds.[...]So when you have a lot of people having different knowledge with data and [they] come together it makes the work easier" (HH)*<br>• *"you don't have the requisites personnel to manage what you have and therefore, you need that extra technical inputs. Once you get that technical input, it will improve the performance, effectiveness and then help with the interventions as well. Because let's take Rabies,[...] if you have a dog bite - the dog looks suspicious, no history, nothing, stray dog. First of all, if the Vet could come in and take the sample from the dog and tell us whether it was a rabid dog or not, it would save us a lot. But ... they don't have the capacity to do it (HH)*
		Stronger Disease Surveillance	• *"We need to collaborate to curb spreading of diseases and outbreaks. Average. [...] I've read somewhere that [for] bird flu, a new variant is now affecting human beings. So if the person goes, or if we get bird flu, and we don't let them know, how will they be ready to track? So as for collaborations, it is very important. And fortunately for us, we the Vets, know that it's important, because we are dealing with zoonoses. And sorry to say but now, I'm happy that the pandemic is*

		and Outbreak Prevention	*happening. And it's all originating from animals. So now, the world will sit up and take this sector serious, because you can't do that in isolation, where the Vet is also doing his own isolation. Yes, if you involve me, anything I see on the field, I put it on the platform that we created" (AH)*
		Enhanced Knowledge, awareness and capacity	• *"We should be able to build capacity [and] learn, from each other." (AH)*<br>• *"Yeah, learning for us as well. Because the more kinds of outbreaks or surveillance work you are doing, the more you are improving your knowledge. Because you can be a specialist but you haven't seen certain [cases]…[so] you collaborate and at the end of the day, you have ideas of how to handle another [case]. Even if they [other sectors] were not there, you would handle it better. [...] [Because of] prior experience ... it's a learning experience." (HH)*
		Resource sharing and mutual benefits	• *"If we could collaborate very well it will help. [...] Like [if] you have a rabies case or even bird flu, it will be proper for the human medics too to be around. So they also do their screening [to see] if the people are affected or not while we also [...] deal with [our part]. Collaboration, is good because I remember there was a time we had a bird flu [case]. We went to the fire service people for their fire tender to do the depopulation. We went to [place] and their tender couldn't work and we went all the way to [2nd place]. So they brought their tender." (AH)*
		Better communication and information flow	• *"Anything that helps the work. We are here to give quality health care services. So, if we realize that what is coming on board will help us give quality health care services, why not? When we collaborate, it makes it easier for all of us because if I'm looking for information, I will not suffer because [the] vet [should be] expecting that I'll call [them]. Because immediately there's an outbreak in the district and [they are] in the district with me [they should] know that 'oh, it means that they will be calling me very soon.' So, [they will] probably start getting [their] information together before I call. So, such collaborations will be good for us because everybody will be ready to provide the information they have. We put it together and analyse it together. So it will be good if they are added on to our reporting channels or to our SOP." (HH)*
		A more holistic cross-sectoral approach to disease control	• *"If we collaborate well, I think that we'll be able to reduce episodes of outbreaks. [...]Imagine if we are doing this collaboration, alright and the people in the wildlife start tracing because they will or should know the wildlife that is there. You know every area and the type of wildlife that is there, so they will know that okay, so this type of wildlife is capable of transmitting, so and so disease but they have been moved from their natural habitat. So where have they moved to? Have they moved further into another forest or [are they] in the closest community? [...] Then they can alert the assembly that 'eii these wildlife were here, we can't find them anywhere. So where are they? Let's see if they're within your assembly.' Then they can also let their environment (health unit) start looking around for that. And if it is found, they can also get in touch with [human] health, [saying] 'we have found so and so,...we have been notified by wildlife [that] they harbour so and so or they are vectors of so and so disease.' [Then human] health can also start community engagement [saying] 'when you're cooking, cook it well, wash your foods, wash your hands, cover your foods, don't leave your food in the open' and all that. That way, we will prevent anyone from getting the disease and then we will not get any outbreak on our hands" (HH)*<br>• *"It will force our various assemblies or whoever's responsible to ensure that our environment is well kept. For instance, we realized that when we are walking around we see cows, goats and all that. Are they being reared where they are supposed to be reared? You and I don't know if there are policies that specifically say that in this area, this is a residential*

			area, do not rear animals here. Do we have anything like that? If we are collaborating with each other, we'll be able to educate each other, animal [healt] can tell assembly that 'this group of animals are vectors for so many people [and] we shouldn't allow them to be at residential areas. Let's get this area for them, where we can make sure that they're kept there.' Then assembly too [will] ensure that their policies or their bylaws concerning some issues are enforced, because now they know the reasons behind it and why those policies should be enforced and it will also help with our environment. Because when these animals are moving around, their droppings are in the community, all sorts of things are in the community. And it will really help with enforcing of bylaws within each of the assemblies." (HH)
