# Supplementary material for: Beyond silos: Drivers and barriers to intersectoral collaboration in zoonotic disease surveillance and response in the Greater Accra Metropolitan Area, Ghana
Source: PLoS One. 2026 Apr 16;21(4):e0347471. doi: 10.1371/journal.pone.0347471 (PMC13086352; doi:10.1371/journal.pone.0347471)
Supplement: Attachment — Submitted filename: Responses to Reviewers.docx [file pone.0347471.s003.docx]

**Responses to Reviewer’s Comments**

**Reviewer 1 Comments**

**We appreciate you taking time out of your busy schedule to review our paper and also providing very detailed constructive criticism/ feedback to improve the work. Please find below proposals to address the various points raised.**

**Abstract**

1. ***Comment*** *- Line 28: I would not use district as a synonym of operational – so better to choose between district and operational, and not keep the two side-by-side*

**Response** - Thank you for this helpful suggestion. We have revised the terminology to use "operational level" consistently, as this accurately captures the multi-level nature of the work performed by our district-level participants, who sometimes operate from community to district levels in their surveillance and response activities. (Line 28)

2. ***Comment*** *- Line 35: when you say “governance and surveillance organization”, is organization applied to surveillance and governance or to surveillance only? In both cases, it sounds a bit strange to me. I wonder if “surveillance governance and organization” would not be more suitable.*

**Response** – Thank you for this insightful observation. We agree with your suggestion and have revised the phrasing to ‘surveillance governance and organization systems’ in the manuscript and subsequent figures. (Line 35)

3. ***Comment*** *-* ***You announce 9 factors, but I count only 8.***

**Response** – Thank you for pointing this out. We have revised the abstract following the structural reorganization suggested in your other comments (22 & 38). The new framework eliminates the counting discrepancy and provides clearer organization of our findings. **(Lines 33 - 39)**

4. ***Comment*** *- Line 39: Please specify the outcomes of what. Collaboration?*

**Response** - Thank you. As stated above, due to the reorganization of our findings we have removed that sentence.

5. ***Comment*** *- Line 45: we usually prefer to use collaboration without ‘s’*

**Response** – Thank you, the ‘s’ has been removed. **(Line 44)**

**Introduction**

6. ***Comment*** *- Line 57: I do not see how limited public health infrastructure can favor spillover. Can you clarify?*

**Response -** Thank you for this important clarification. You are correct that limited public health infrastructure does not directly favor spillover events. We have revised the sentence to clarify that while frequent human-animal interactions and weak surveillance systems create conditions for spillover, limited public health infrastructure facilitates the spread and impedes effective response. **(Line 56-57)**

7. ***Comment*** *- Line 59: 28 934 is not really an approximation and I am a bit skeptical about our capacity to count cases so precisely, especially in SSA where surveillance systems are weak … I would suggest “approximately 30 000”.*

**Response -** Thank you. We agree that the level of precision implied by “28,934” may not be appropriate given the limitations of surveillance systems in sub-Saharan Africa. We have revised the sentence to read: **“Over the past two decades, zoonotic diseases have caused approximately 30,000 human infections in sub-Saharan Africa.” (Line 58)**

8. ***Comment*** *- Line 62: I think you could improve your definition of One Health. It is an integrated approach but it does not integrate healths – it recognizes that they are interconnected, which require “integrated” actions.*

**Response -** Thank you for this important clarification about the One Health definition. You are correct that One Health does not integrate the different health sectors themselves, but rather recognizes their interconnection and promotes integrated actions across them. We have revised the definition to accurately reflect this conceptual distinction. (Line 62)

9. ***Comment*** *- Lines 63-65: I do not understand this sentence. Please clarify.*

**Response -** Thank you for highlighting this unclear sentence. Our intention was to convey two points: first, that intersectoral collaboration is essential for effective disease surveillance; and second, that this collaboration is frequently hampered by significant challenges such as governance issues, fragmented systems, resource limitations, and poor communication.We have revised it to improve clarity and readability. (Line 64-66)

10. ***Comment*** *- Line 67: I would suggest to change “district (operational)” for “sub-national”. And then specify that you are working at the level of the district (and why) in the methodology part.*

**Response -** Thank you for the suggestion to use "sub-national." However, we believe "operational level" is more appropriate for our study context (Line 67). "Sub-national" would be too broad as it encompasses regional, district, and sub-district levels, whereas our study specifically focuses on district-level officers and their operational activities. While these officers are positioned at the district level, their surveillance and response work spans from community to district levels, making "operational level" the most accurate descriptor. We have clarified this distinction in the methodology section. (Line 117- 119)

11. ***Comment*** *- Line 75: I would suggest to delete “or fails” that suggest that you anticipate that it is not working*

**Response -** Thank you for this helpful suggestion. We agree that the phrase may imply a presumption of dysfunction. To maintain a more neutral tone, we have removed it. (Line 76)

12. ***Comment*** *- Line 79. I suggest to add “for zoonotic diseases” after systems.*

**Response –** Thank you. It has been added (Line 80)

**Methods**

13. ***Comment -*** *Lines 101-102. Please describe briefly these criteria for the reader, so he/she can assess the potential impacts on the study results*

**Response -** Thank you for this request. We have added a brief description of the larger research project's objectives to help readers understand the study selection criteria and assess potential impacts on results. We explain that the project assessed Ghana's zoonotic disease surveillance and response systems (ZDSR) with multiple objectives, including sectoral evaluation (explaining uneven district participation), intersectoral collaboration examination, and barrier identification for One Health implementation (Lines 95-104).

We have also cited our recently published paper from this project (Dsani et al., 2025, One Health), which describes the current intersectoral collaboration environment. We wish to clarify the relationship between the two manuscripts for transparency:

**Published manuscript (Dsani et al., 2025):** Uses content analysis to descriptively characterize current collaboration patterns (who collaborates, on which diseases, in which surveillance activities)

**Current manuscript:** Uses thematic analysis to identify factors that drive and hinder collaboration, analyzing barriers, facilitators, and motivations

Both manuscripts use the same interview dataset but employ different analytical approaches to address distinct research questions, with no overlap in results or discussion.The published work describes collaboration patterns; this manuscript explains why these patterns exist. Each represents an independent contribution to One Health scholarship.

14. ***Comment -*** *Line 119: the number of invitations sent is missing – did everyone accept to be part of the interview? if not, why?*

**Response -** Thank you for this important clarification. We have added details about invitation and response rates. Initial invitations were sent to 32 district heads (16 human health, 16 animal health), with high acceptance rates (15/16 and 16/16 respectively). We clarify that fewer interviews were ultimately conducted due to data saturation, scheduling conflicts, and time constraints, and explain the flexible participation approach we used. (Line 131 - 140)

15. ***Comment*** *- We are also missing the distribution of participants across the 16 districts to assess the level of representativity of each district.*

**Response -** Thank you for this suggestion. We understand the importance of demonstrating representativeness. However, providing district-level distribution data would significantly compromise participant confidentiality in our study context. The animal health sector has very small numbers of practitioners at the district level - in many cases only 1-2 per district - and since we interviewed complete teams in most districts, a table showing participant counts would essentially create a map that readers familiar with the animal health sector could use to identify specific districts. For example, if a table showed "District X has 4 animal health participants," colleagues in the sector would immediately know which district this refers to, as very few districts have 4 animal health officers. Combined with contextual details in quotes, this would compromise the anonymity we promised participants.

We provide narrative information confirming that participants were recruited from across all 16 districts, with variation in numbers reflecting differences in sector sizes, purposive sampling of key ZDSR stakeholders, and one district's involvement in another study component (Lines 157-164). We believe this approach balances the need to demonstrate broad geographic representation with our ethical obligation to protect participant confidentiality in a small, highly interconnected professional community.

16. ***Comment -*** *Lines 137-138: ECoSur details organizational (at governance and operational level) and functional attributes (and not functional and governance attributes)*

**Response –** Thank you. The corrections have been effected. (Line 167 - 170)

17. ***Comment -*** *Line 138. The “.” should be replaced by a “;”.*

**Response -** Thank you for pointing this out. We have replaced the period with “; and” to ensure correct punctuation and maintain the parallel structure of the listed frameworks. (Line 170)

18. ***Comment -*** *Line 154: I do not get how multiple readings ensure accuracy; and accuracy of what? please explain.*

**Response -** Thank you for this clarification. Since the 1^st^ author conducted 64 out of the 66 interviews themselves (with a research assistant conducting 2 interviews), they were already familiar with the data content. The multiple readings were specifically to ensure transcription accuracy by checking the transcripts against the original audio recordings. We have revised the text to clarify this distinction. (Line 189 - 190)

19. ***Comment -*** *I suggest to put together paragraph lines 80-82 with paragraph lines 91-96, and move the contents of lines 83-90 to the method part.*

**Response -** Thank you for this helpful structural suggestion. We agree that combining the study objectives (lines 80-82 and 91-96) creates better flow in the introduction (now on Line 81- 90).We have moved the stakeholder definitions to the methods section where they provide important context for understanding our participant selection (Line 108-111). We have also moved the methodological justification for using qualitative approaches to the methods section, where it better explains our research design choices (Line 122 - 124).

20. ***Comment -*** *It is usually advised that the coding is done by two people to improve accuracy. If you did not do it, you need to discuss this potential bias in the limits of the method*

**Response -** Thank you for this valuable comment regarding coding practices. We agree that dual coding by two independent researchers is ideal for enhancing accuracy and reducing bias in qualitative analysis.

In our study, the primary coding was conducted by the first author. To ensure reliability and consistency, a second researcher independently re-coded three transcripts to cross-check the identified themes and verify application of the analytical framework. Additionally, four transcripts were coded collaboratively by both researchers to clarify emerging themes and refine the coding scheme through discussion. This verification process confirmed the consistency of our coding approach and the robustness of derived themes.

We acknowledge in our limitations section (Lines 831-838) that while we implemented measures to enhance trustworthiness, dual coding throughout the entire analytical process would have been ideal to further minimize potential single-coder bias in theme development and data interpretation.

21. ***Comment -*** *Lines 171-178: the contents of these 2 paragraphs should be moved to Study design and Data collection.*

**Response -** Thank you for this suggestion. We have moved the contact and recruitment procedures to the "Study design and participant selection" section and better integrated it with the surrounding paragraph to improve the flow (see Lines 131 - 135). We have also moved the interview consent and recording procedures to the "Data collection" section where they are more appropriately placed (see Lines 179 - 182).

**Results**

22. ***Comment -*** *Why did you address motivation, expectations, and perceived benefits separately from factors driving and hindering collaboration?
    To me they are also factors that influence collaboration. And you present them as such at the beginning of the discussion (lines 621-622).
    Please clarify and explain how everything is linked, or introduce them as specific themes in part A. and merge everything together*

**Response -**

**Thank you for this valuable comment. We agree that motivations, expectations, and perceived benefits all influence collaboration and should be integrated rather than treated separately. We have thus revised the Results section to reflect this** (Lines 226 - 683)**.**

**Specifically, we have:**

- **Reorganized our results into 'Factors Influencing Intersectoral Collaboration in ZDSR'** with two categories: **Individual factors** (relationships, personal initiative, motivations, perceptions of superiority, shared goals), and **Structural factors** (governance, visibility, workforce availability, knowledge, and resource constraints). Positive collaborative outcomes fall within both individual and structural factors.
- **Integrated ‘motivations’** into the ‘individual factors’, emphasizing that they represent participants' underlying incentives for engaging in collaboration.
- **Renamed ‘perceived benefits’** to ‘Positive collaborative outcomes’ and placed it as a factor that falls under both individual and structural factors.
- **Separated ‘general expectations for future collaboration’** as stakeholder aspirations to clearly distinguish aspirational views from demonstrated influences on current collaboration.

**This restructuring creates a more cohesive framework linking motivations, experiences, and expectations as interconnected influences on collaboration.**

23. ***Comment -*** *Lines 183: why perceived? they were suggested by participants as factors? It is not what I understood in the methodology. They are perceptions of interviewees that you have identified, with your coding process, as being determinants to collaboration; but to me, they are not perceived factors per se (which suggests that interviewees identified themselves the factors)*

**Response -** Thank you for this helpful comment. We initially used the word “perceived” to emphasize that the factors identified in this study were based on the lived experiences and perspectives of the interviewed surveillance actors, rather than objectively measured determinants. Our intention was to distinguish these from causal claims or externally validated drivers. However, we understand how the use of “perceived” in the heading may imply that participants themselves explicitly identified and labeled these as “factors,” which was not always the case. To avoid confusion, we have removed the term and simply described it as ‘factors influencing collaboration’ also while retaining the necessary context in the body of the text to clarify that these findings are grounded in participants’ descriptions, as interpreted through thematic analysis. (Line 227 – 228 and 235 - 237).

24. ***Comment -*** *Line 186. I do not think that an expectation can be perceived*

**Response -** Thank you for this observation. We agree that the phrasing was unclear and have revised the subheading to: “Aspirational expectations” to more accurately reflect the intent. (Line 233 - 235).

25. ***Comment -*** *Line 189: I do not get why assigning a unique identifier to participant will allow to link quotes to specific individuals. And I think the reader needs this information to assess the diversity of individuals you are quoting. It would be even better if you could give a code to the district where the interviewee is working to assess the representativeness of the districts*

**Response –** Thank you very much for this important suggestion. We fully agree that demonstrating diversity is essential in qualitative reporting. However, in our study context, assigning unique participant identifiers (e.g., HH1, AH2) or district codes would significantly increase the risk of deductive disclosure.

Many of the most analytically valuable quotes are also highly specific, such as describing the destruction of a clinic, being introduced to a public health team during COVID, meeting a veterinarian for the first time at a budget meeting and later inviting them to collaborate, or treating animals of the assembly coordinator. Readers familiar with the local context could easily identify the individual behind such quotes. The risk is that once a participant is identified through one distinctive quote, any identifier system would allow all other comments from that participant, including sensitive critiques of other sectors, to be linked to them. This risk is particularly acute in the animal health sector where most districts have only a single animal health professional, making the connection between quotes instantaneous and unavoidable. Any form of identifier, whether district codes, gender, professional role, years of experience (in surveillance or in the district), work level, or other demographic characteristics collected, would create this same linkage problem, effectively deanonymizing participants.

This issue is even more pronounced for wildlife health actors (n = 3, all at regional/national level) and so to eliminate any risk of disclosure, we decided not to include their direct quotes. Instead, we reflected their perspectives through cross-cutting themes raised by other sectors.

We also took steps to ensure that the findings were not skewed by overrepresentation from any single location. Although one district contributed the largest number of human health participants due to its involvement in another objective of the broader research project, we did not include any quotes from human health informants from that district to illustrate any theme in this manuscript. Overall, the quotations used to illustrate themes in this manuscript were drawn from participants across 15 of the 16 districts, reflecting wide geographic coverage and diversity of perspectives.

To balance transparency with confidentiality, we report quotes by sector only (HH = human health, AH = animal health, WI = wildlife health). Readers can assess sample diversity through the narrative description of participant distribution in the Methods section (Lines 157-164), which confirms recruitment across all 16 districts and all work levels without providing identifying details. We have moved the information about participant anonymity from the Results section and expanded it into a comprehensive 'Participant Confidentiality' subsection under Ethical Considerations (Lines 211-233). This section explains our sector-only labeling approach, the rationale behind avoiding all demographic identifiers, and our specific quote selection decisions.

Additionally, the Data Analysis section clarifies that all themes reported in the manuscript were derived from multiple participants, ensuring that no single theme is based solely on one individual’s perspective (Lines 200–202). We believe this approach provides sufficient transparency while fully safeguarding participants’ anonymity.

26. ***Comment -*** *Lines 196-197: you said that the factors are grouped into 2 categories (structural and individual) but then these categories are not used in the description of the factors. In addition, in the abstract you mention sociocultural, structural and governance factors, but you do not identify these groups in the Result section. You need to harmonize across the text. Then in the discussion part, you mention again the structural and individual factors but we do not know which ones fall into these 2 categories.*

**Response -** Thank you for this observation. We have harmonized terminology throughout the manuscript to ensure consistency between the abstract, results, and discussion sections. We now use the same two-category framework (individual factors and structural factors) across all sections. We have also revised the abstract to remove 'sociocultural' terminology (Line 40). that was not clearly defined in the Results section, ensuring that all category labels align with our analytical framework.

**Discussion**

27. ***Comment -*** *In the abstract, you mention that levers for better collaborations are stronger governance, equitable partnerships, and realistic alignment of stakeholder expectations. However, in the discussion, we do not identify these 3 main levers.*

**Response -** Thank you for this observation. We have addressed these three key areas throughout our discussion section, though not explicitly labeled as "levers." We have now strengthened the connections between our findings and these implications:

- **Equitable partnerships:** In the discussion of informal networks and personal initiative (Lines 733-741), we have added analysis emphasizing that relying on individual assertiveness to overcome structural barriers places unfair burdens on marginalized sectors. We now explicitly argue for intentional institutional design that distributes collaborative authority through formalized roles rather than expecting individuals to prove their worth repeatedly.
- **Stronger governance:** In the structural barriers section (Lines 750-757), we discuss how weak governance creates fragmentation and inconsistency. We have strengthened this by explicitly stating that governance reform must extend beyond policy adoption to operational implementation, ensuring collaborative structures function consistently regardless of political cycles or individual champions.
- **Realistic alignment of stakeholder expectations:** This is addressed comprehensively in our dedicated section on expectation-reality gaps (Lines 813-834), where we analyze how misaligned expectations create barriers and discuss the need for establishing clear, realistic collaborative objectives from the outset.

These revisions ensure that the implications stated in our abstract are clearly articulated throughout the discussion section.

28. ***Comment -*** *Lines 633-634. Please explain and not only make references to other articles.*

**Response -** Thank you for your feedback. We have revised the discussion section to provide a more detailed explanation of how informal relationships introduce inconsistencies, unsustainability, power imbalances, and exclusivity. As revised in the manuscript, we clarify that these relationships, unlike formal structures, lack standardized procedures and are vulnerable to changes in personal dynamics or staff turnover. This makes collaboration inconsistent and unsustainable. We also explain that because informal networks are often built on pre-existing social ties, they can reinforce existing hierarchies and limit participation to a select group, thereby creating power imbalances and exclusivity regardless of expertise. We appreciate you pushing us to more clearly articulate this crucial point. (Lines 712 - 723)

29. ***Comment -*** *Lines 655-657. You refer to participants discourse but you had references to other studies. This is inconsistent.*

**Response -** Thank you for identifying this inconsistency. We have revised the sentence to clarify that while our participants reported financial constraints as barriers (as presented in the Results), the literature supports that such resource disparities create power imbalances and dependency relationships. The revised sentence now reads: ‘Financial constraints create power imbalances as disparities in resource allocation make certain sectors dependent on better-funded institutions for logistical support.’ (Lines 743 - 745) This removes the ambiguity about whether we are reporting participant discourse or citing supporting literature.

30. ***Comment -*** *Lines 658-659. I do not see the link between the profit-driven dimension of collaboration and resource disparities among stakeholders. Please specify.*

**Response -** We recognize this connection was unclear. We have restructured the paragraph to distinguish between two related but distinct financial barriers: (1) resource disparities creating dependency and power imbalances, and (2) financial mistrust arising from perceptions of profit-driven motives and fears of fund misappropriation. The revised text now states: 'Financial mistrust is known to discourage collaborative initiatives [50, 51], a dynamic clearly present in this study where participants avoided collaboration rather than risk resource exploitation.' This clarifies that we are interpreting our participants' experiences through established literature on financial mistrust in partnerships. (Lines 745 - 749)

31. ***Comment -*** *Lines 678-683. I do not see how ideas in this paragraph articulates with each other. How will mutual respect and engagement contribute to raise awareness and access? It is also partly redundant with the contents of the paragraph before.*

**Response -** Thank you for this observation. You are correct that the connection between One Health education and the preceding discussion of workforce availability and sectoral visibility was unclear and the content was redundant. We have removed this paragraph. Given the extensive scope of factors identified in our results, we focused our discussion on the most critical structural barriers (financial constraints, governance weaknesses, and workforce/visibility issues) that most directly impeded collaboration. While knowledge and training emerged as factors in our results, we prioritized discussing the structural conditions that create the context within which all other factors operate.

32. ***Comment -*** *Lines 684-690 seem redundant with what has been said before (sectoral visibility)*

**Response -** Thank you for this observation. We agree that this paragraph's placement within the structural barriers section created confusion. We have relocated it to serve as part of the concluding paragraph of the entire discussion section. In this position, it effectively synthesizes the relationship between individual agency and structural constraints discussed throughout all subsections, reinforcing our key finding that individual adaptations, while important, cannot substitute for systemic reform. (Lines 825 - 834)

33. ***Comment -*** *Lines 754. You mention that focusing on Ghana prevents the generalization to other LMICs but in the abstract you state that you results are applicable to other LMICs. Please be clearer on what can be generalized and what cannot.*

**Response -** We appreciate the reviewer's observation regarding the need for greater clarity on the generalizability of our findings. We agree that our initial wording created an ambiguity between the context-specific nature of our study and the broader applicability of its insights.

To address this, we have revised the abstract (Lines 41 - 45) and the limitations section (Line 836 - 864). Our intention is to clarify that while the in-depth operational-level insights are rooted in the Ghanaian context, making direct generalization of all detailed findings challenging, the broader categories of factors influencing intersectoral collaboration (e.g., individual relationships, resource constraints, and governance structures) and the overall principles for strengthening One Health are highly relevant and transferable to other LMICs. We believe the revised wording now explicitly distinguishes between context-specific observations and more broadly applicable lessons, providing a more nuanced understanding of our study's scope and implications.

**Conclusion**

34. ***Comment -*** *In addition, you have worked on a limited number of districts in a single region. Can you even generalize your results to Ghana? Please discuss this point.*

**Response -** Thank you for this important observation. You are correct that we studied GAMA (Greater Accra Metropolitan Area), not all of Ghana, and should not claim generalizability beyond this region. We have revised the conclusion to specify 'in Ghana's largest metropolitan area' rather than 'in Ghana' (Line 868). We have also expanded the limitations section to clarify that while we sampled 16 of GAMA's 25 districts (more than half), findings reflect one urban metropolitan region and may not generalize to rural or other regional contexts in Ghana without further research (Lines 850-857).

Additionally, as noted in our response to comment 33 about generalizability to other LMICs (Lines 41-45 in abstract), we have clarified that while the types of factors identified likely resonate across different contexts, their specific manifestations and relative importance may vary depending on local circumstances.

35. ***Comment -*** *Lines 771. This is not the first study highlighting the role of informal networks, personal initiative, and structural constraints in intersectoral collaboration. This has been described in some of the literature cited in this study;*

**Response –** Thank you for this comment. We agree that the concepts of informal networks, personal initiative, and structural constraints in intersectoral collaboration have been highlighted in previous literature. Our intention was not to claim novel discovery of these general factors themselves.

We have revised the text at Line 869 - 871 to clarify our contribution. Our study's uniqueness lies in providing rich, context-specific operational-level insights into *how* these factors specifically manifest and critically shape intersectoral collaboration within the zoonotic disease surveillance and response system in Ghana. By offering detailed empirical evidence from this particular setting, our research aims to complement and deepen the existing understanding of these crucial elements in One Health implementation.

36. ***Comment -*** *Line 779. Inconsistent with the limit you have mentioned above, about the generalization of your work (see my comment above).*

**Response -** Thank you for highlighting the inconsistency in our conclusion regarding generalizability, which echoes your previous comments (33 & 34). We agree that the statement at Line 779 needed to be more aligned with the limitations of the study and our intended message on transferability.

We have revised the conclusion section to reflect a more precise understanding of how our findings can inform One Health implementation beyond Ghana. The revised text now emphasizes that our study provides Ghana-specific evidence that highlights fundamental operational-level challenges and facilitators, and that these findings offer transferable lessons and inform the development of targeted strategies for other LMICs. This revision aims to ensure consistency across the manuscript regarding the nuances of generalizability and to clarify that our work offers valuable insights applicable to similar resource-limited settings. (Line 889 - 887).

37. ***Comment -*** *Line 780. Only public health outcomes? how about animal health outcomes? Economic outcomes? Social outcomes?*

**Response -** We thank the reviewer for this important comment. We fully agree that the benefits of strengthening intersectoral collaboration within a One Health framework extend beyond public health. We have revised the final sentence of the conclusion to reflect this broader impact, now stating that such collaboration will improve public, animal, and wildlife health outcomes, and foster more coordinated and resilient response systems that can also yield socio-economic benefits. This aligns with the comprehensive nature of the One Health approach and the intersectoral engagement discussed throughout our manuscript. (Line 886).

**General Comments**

38. ***Comment -*** *Conversely, the format of the results section should be deeply revised. The thematic analysis allows you to identify relevant themes related to your research questions. But, when describing the results, and especially in a scientific article, you need to bring back everything together in a well-articulated text. No need to clearly state Theme 1, sub-theme 1, etc. They are not themes anymore, they are what you are willing to describe, eg factors. You can have one separate part for each key factors and then describe the sub-themes in these parts, mentioning if they are acting as barrier or driver. Then for parts B, C, and D, you should write a descriptive analysis of your factors and not provide the results in a table. You can refer to other articles in that field to see how to better structure your result section. I think this should allow you to group some of the sub-themes together among themes and across themes, among parts and across parts (see my comment in the Results sections about merging the 4 parts as motivation, expectations and benefits can also be considered as factors influencing collaboration). Some seem redundant.*

**Response -** Thank you for this detailed guidance on academic writing style for qualitative research. We have revised the Results section to follow established conventions for presenting qualitative findings:

- **Eliminated mechanical "Theme 1, Sub-theme 1" formatting** and restructured around key factors described in flowing narrative paragraphs
- **Integrated quotes naturally within descriptive text** rather than presenting them in tables or bullet points
- **Clearly indicated whether each sub-theme acts as a facilitator, barrier, or both** within the narrative flow
- **Modeled the presentation style on established qualitative collaboration studies** to ensure appropriate academic formatting

This revision creates a more sophisticated, well-articulated presentation that meets the standards for qualitative research reporting in peer-reviewed journals.

39. ***Comment:*** *In addition, it is difficult to see how some verbatims are illustrating the text (for instance, verbatim lines 309-313).*

**Response -** Thank you for highlighting this. The verbatims illustrate mistrust about fund misuse, though the connection may not be immediately apparent. In this context, "writing a memo" refers to submitting joint funding requests to the district assembly. The first participant expresses reluctance to do so because they believe environmental health partners would "take the money" (receive the funds) but "you'll not get the money to do the work" (the funds wouldn't be used for the intended collaborative activities). The second participant reinforces this concern about fund appropriation. To clarify this for readers, we have revised the lead-in text to better contextualize both supporting quotes and make the connection to mistrust about fund misuse more explicit. (Lines 402 - 413)

40. ***Comment:*** *There are a lot of redundancies and inconsistencies in the discussion part. I suggest to restructure this part and make your message clearer. There are very interesting and innovative ideas but they are drowned out by repetition of things already said elsewhere in the discussion section or even in the results section. I have provided one example in the comment below, but it applies to other parts of the discussion.*

**Response -** Thank you for this valuable feedback. We agree that the discussion contained redundancies that detracted from our key messages. We have substantially restructured this section to eliminate repetition and present our arguments more clearly and concisely.

Specifically, we have:

- **Consolidated overlapping content** by removing sentences that restated points already made within the same section or across different sections
- **Reorganized the discussion flow** to follow a logical progression: individual factors (informal networks and motivations), structural factors, positive collaborative outcomes, and expectation-reality gaps
- **Added a new section on positive collaborative outcomes** as reinforcing mechanisms, which was a key finding that deserved dedicated discussion
- **Removed repetition between results and discussion** by ensuring the discussion interprets and contextualizes findings rather than re-describing them

These revisions have reduced redundancy while strengthening the clarity and impact of our key contributions, particularly around the role of informal networks, self-preservation motivations, and the feedback loops created by successful collaboration.

41. ***Comment:*** *I am also surprised that you address the need to align expectations among stakeholders from the different sectors on one hand, but refer to collaboration outcomes only for public health on the other (lines 29-30). One Health is seeking shared benefits across the human, animal and ecosystem health. Supporting this anthropocentric definition of One Health – as done in this manuscript- is contributing to maintain expectation gaps across sectors.*

**Response -** Thank you for this observation. We have revised the abstract to reflect that One Health collaboration seeks shared benefits across human, animal, and wildlife health rather than framing outcomes narrowly as 'public health outcomes.' The revised text in the abstract now reads: '...providing insights to strengthen cross-sector collaboration and improve health outcomes across human, animal, and wildlife sectors. (Lines 29 - 30). We also revised a sentence in the introduction that had the same phrasing. (Line 87)

42. ***Comment:*** *In general, you need to write more and avoid making lists with bullets or tables with a lot of text that is difficult to read*

**Response –** We appreciate the feedback regarding the presentation of our manuscript. We understand the concern that extensive use of bullet points and text-heavy tables can hinder readability. We have revised the manuscript to reduce the use of bullet points and present information in prose format instead. Specifically, we have converted the participant description section from bullet points to narrative paragraphs that provide the same detailed information in a more readable format. We have also revised our ‘Limitations of the Study section’ by converting the previous bulleted list into a more cohesive paragraph format. We have also reviewed other sections to minimize excessive use of lists or fragmented text where continuous prose would be more appropriate.

43. ***Comment:*** *You use “cross-sectoral”, “cross sector” and “inter-sectoral” to characterize collaboration in your manuscript. If you use them interchangeably, I would suggest to opt for one and keep it all along the manuscript. If they have different meaning, please specify.*

**Response –** Thank you for the very helpful comment. We have addressed the inconsistency by standardizing the terminology to ‘intersectoral’ throughout the manuscript.

44. ***Comment:*** *I would suggest to keep intersectoral collaboration in full, instead of using an acronym.*

**Response:** The acronym ‘ISC’ has been changed to ‘intersectoral collaboration’ all over the manuscript

45. **Other changes -**

- Figure 1 has been updated to reflect the new results structure and a caption for it has been included in the manuscript (Lines 603 -613)

**Reviewer 2 Comments**

Thank you very much for reviewing our work and for your feedback. We have tried our best to address your suggestions.

46. ***Comment -*** *I consider this manuscript to be of interest to machine learning researchers in biological systems as well as readers of this journal. As such, I am generally supportive of publication with a few minor comments. While the authors focus on reflexive thematic analysis, there have been recent studies using advanced machine learning methods, such as t-SNE method, which can be used efficiently for classification as well as detecting trends in biochemical systems, which should be noted: Environ. Sci. Technol. Lett. 2023, 10, 1017–1022 and Mar. Genom. 2020, 51, 100723. In particular, these prior studies showed that t-SNE can give classification of results that could help diagnose trends in biochemical systems. I am not asking the authors to carry out such t-SNE calculations, but these recent applications of t-SNE should also be noted.*

**Response -** We appreciate the reviewer's positive assessment and support for our manuscript. We're also grateful for their acknowledgement of the paper's potential interest to machine learning researchers.

However, we'd like to respectfully clarify that our study focuses on health systems research, specifically exploring the qualitative aspects of intersectoral collaboration within zoonotic disease surveillance. While the cited machine learning methods are indeed powerful for analyzing pure biological or biochemical systems, our research deals with complex human interactions, governance, and social dynamics, which are distinct from the types of data typically analyzed by t-SNE for classification or trend detection in biochemical contexts. Our chosen methodology, reflexive thematic analysis, was specifically selected to deeply understand these nuanced human experiences, which are central to health systems.
