# Supplementary material for: Beyond silos: Drivers and barriers to intersectoral collaboration in zoonotic disease surveillance and response in the Greater Accra Metropolitan Area, Ghana
Source: PLoS One. 2026 Apr 16;21(4):e0347471. doi: 10.1371/journal.pone.0347471 (PMC13086352; doi:10.1371/journal.pone.0347471)
Supplement: Attachment — Submitted filename: Review_V2.docx [file pone.0347471.s004.docx]

Thank you for your detailed explanations. I still have a few comments, which I outline below.

**Abstract**

*2.* ***Comment*** *- Line 35: when you say “governance and surveillance organization”, is*

*organization applied to surveillance and governance or to surveillance only? In both cases, it*

*sounds a bit strange to me. I wonder if “surveillance governance and organization” would*

*not be more suitable.*

**Response** – Thank you for this insightful observation. We agree with your suggestion and

have revised the phrasing to ‘surveillance governance and organization systems’ in the

manuscript and subsequent figures. (Line 35)

Sorry, but it is still not clear to me. I suggested “the governance and organization of surveillance”. What do you mean by organization systems? system of what?

In addition, you announce factors influencing disease surveillance and response, but mention only surveillance here.

*5.* ***Comment*** *- Line 45: we usually prefer to use collaboration without ‘s’*

**Response** – Thank you, the ‘s’ has been removed. (Line 44)

But, it still remains in other parts of the manuscript

**Introduction**

***6. Comment*** *- Line 57: I do not see how limited public health infrastructure can favor spillover.*

*Can you clarify?*

**Response -** Thank you for this important clarification. You are correct that limited public

health infrastructure does not directly favor spillover events. We have revised the sentence to

clarify that while frequent human-animal interactions and weak surveillance systems create

conditions for spillover, limited public health infrastructure facilitates the spread and impedes

effective response. (Line 56-57)

Still not clear to me. How weak surveillance systems creates conditions for spillover? The limited public health infrastructure facilitates the spread of what? I would move the weakness of surveillance to an additional factor that facilitate that impeded effective response.

Lines 75-77 soften the statement of lines 69-71 and should be put side-by-side.

Not sure that lines 71-74 are bringing relevant information in the context of this study.

Lines 77-78. Why is it specifically crucial to understand collaboration dynamics when there is a high zoonotic disease burden and under-resourced health systems? The causal link is not obvious to me

Lines 84-90 sound more like a conclusion than an introduction to the study that will be presented after. Should be rephrased.

**Methods**

Line 100: what do you mean by “which accounts for the higher participant numbers in one district” ?

Line 103: what do you mean by component? Before, you are talking about objectives

Lines 139-140. They agreed, but were they ultimately interviewed?

***15. Comment*** *- We are also missing the distribution of participants across the 16 districts to*

*assess the level of representativity of each district.*

**Response -** Thank you for this suggestion. We understand the importance of demonstrating

representativeness. However, providing district-level distribution data would significantly

compromise participant confidentiality in our study context. The animal health sector has

very small numbers of practitioners at the district level - in many cases only 1-2 per district -

and since we interviewed complete teams in most districts, a table showing participant counts

would essentially create a map that readers familiar with the animal health sector could use to

identify specific districts. For example, if a table showed "District X has 4 animal health

participants," colleagues in the sector would immediately know which district this refers to,

as very few districts have 4 animal health officers. Combined with contextual details in

quotes, this would compromise the anonymity we promised participants.

We provide narrative information confirming that participants were recruited from across all

16 districts, with variation in numbers reflecting differences in sector sizes, purposive

sampling of key ZDSR stakeholders, and one district's involvement in another study

component (Lines 157-164). We believe this approach balances the need to demonstrate

broad geographic representation with our ethical obligation to protect participant

confidentiality in a small, highly interconnected professional community.

Sorry, but it is not clear to me how you made your sampling. On one hand you are talking about purposive and convenience sampling, and then you say that you interviewed complete teams in most districts. In addition, you talk about 19 districts first, and then you say that participants were distributed across all 16 districts Be more precise about the sampling strategy used at each stage (selection of district, selection of participants).

Lines 156-157 should be placed at the end of the previous paragraph

Lines 158-160: I do not understand the causal link in this sentence

I am not sure we need lines 160-164

Line 200: I would add discourse after participants. What do you think?

Lines 222-223 – why do you have selected no verbatims for one district?

**Results**

I find the text difficult to read because all of the quotes that interrupt the text. May be, you can embed them in the text. Example: Personal rapport between actors was one of the strongest enablers of collaboration. Friendly relationships fostered trust, encouraged communication, and facilitated informal information exchange. As one animal health informant explained: “It’s because we have the rapport...” (AH)

I would also suggest not to put one verbatim after each statement, but to articulate some statements together and choose the best illustrative verbatim (and the easier one to understand for people not familiar with the context, because a lot of verbatims are difficult to understand)

It would be helpful to define what an assembly coordinator is as they are mentioned in a lot of verbatims

Line 237-238. What is the difference between quotes integrated in the text and the ones provided in the supplementary file 1?

line 246: you need to better explain this quote. What is “it” referring to?

Lines 301-303 – It is not clear to me what you want to stress out. The professional recognition of who by whom create motivation of who?

Lines 304: Self-protection is illustrated later lines 312-314. So why mentioning it here?

Lines 310. What is NADMO? If we do not now, we do not see the collaboration here

Lines 341-345. I do not see how this verbatim illustrate alignment between priorities of actors. The AH actor is talking about views, not priorities. This is more in line with the fact they are both new? Cf lines 251-256

Lines 556-557. Lack of exposure to what? We need to better see the link with the theme “knowledge and training”

***22. Comment -*** *Why did you address motivation, expectations, and perceived benefits separately from factors driving and hindering collaboration?*

*To me they are also factors that influence collaboration. And you present them as such at the*

*beginning of the discussion (lines 621-622).*

*Please clarify and explain how everything is linked, or introduce them as specific themes in*

*part A. and merge everything together*

**Response -**

Thank you for this valuable comment. We agree that motivations, expectations, and

perceived benefits all influence collaboration and should be integrated rather than treated

separately. We have thus revised the Results section to reflect this (Lines 226 - 683).

Specifically, we have:

▪ Reorganized our results into **'Factors Influencing Intersectoral Collaboration in**

**ZDSR'** with two categories: **Individual factors** (relationships, personal initiative, motivations, perceptions of superiority, shared goals), and **Structural factors**

(governance, visibility, workforce availability, knowledge, and resource constraints).

Positive collaborative outcomes fall within both individual and structural factors.

▪ Integrated **‘**motivations’ into the ‘individual factors’, emphasizing that they

represent participants' underlying incentives for engaging in collaboration.

▪ Renamed ‘perceived benefits’ to ‘Positive collaborative outcomes’ and placed it as a

factor that falls under both individual and structural factors.

▪ Separated ‘general expectations for future collaboration’ as stakeholder aspirations

to clearly distinguish aspirational views from demonstrated influences on current

collaboration.

This restructuring creates a more cohesive framework linking motivations, experiences, and expectations as interconnected influences on collaboration.

Ok, but you should mention in the introduction of the Results’ section the positive outcomes, the facts that they may be Individual of Structural factors and that’s why you address them separately

**Discussion**

There is still work to be done on the discussion. It could be more concise and better articulated. In addition, there are often very broad and somewhat vague recommendations, and we would like the authors to go a little further in explaining how these recommendations could be implemented in the context of Ghana and in light of their work.

Line 723. What do you mean by inequities? Is that linked to power imbalances that you are mentioning below? If yes, the link between the two paragraphs should be clearer.

I can see that you are also mentioning inequities line 732

Lines 724. I would take the opportunity of this section to discuss the fact that participants perceived that medics trained in Public health are more collaborative than the ones who are not

Line 726. I understand your point but, to me, power imbalance is real as the human health sector is usually more well-resourced than the animal health sector.

Line 737-740. But what do you propose for this to happen? We have seen that almost all African countries have now One Health Platforms but they are barely non functional and inequities as well as power imbalances persist. So how can we make OH institutionalisation working? Some studies show that too rigid institutional mechanisms also hamper collaboration because people see a risk to loose their autonomy and to be “forced” to do things they do not want to do (such as sharing data).

In addition, I am not sure that institutionalization is synonymous of equity. We have seen that institutionalization may also reinforce inequity. For instance, all international regulations which are mainly focusing on priorities of the “North” shape how funding is allocated to countries and to specific national counterparts. However those countries might have other priorities than the ones the international standards are focusing on and the funding mechanisms may create competition between national actors to accede fundings

Line 750. Poor enforcement of what?

Line 754-756. Ok, but how to achieve this? In the previous paragraph, you are calling for more institutionalisation, but now you say that it is not enough for collaboration to be effective.

You need to discuss these two points together and propose concrete solutions to that or at least way of improvement.

Line 769. I do not understand what this paragraph is adding to what is described previously. You mention physical proximity but then you are mainly refering to visibility, and are redundant with the previous paragraph. These two paragraphs should be merged and ideas better articulated

Lines 825-827. To my knowledge, it has been already addressed in the literature. See for instance: <https://doi.org/10.1186/s12889-022-13878-3>.

Lines 829-833. I don't see how you are proposing a roadmap; at best, you are proposing avenues for reflection and points to consider.

Lines 824- 833. I do not see how this paragraph relates to Bridging Expectation–Reality Gaps. It looks more like a summary of what you have highlighted before. It could be merged with your paragraph about the potential generalisation of your results to Ghana and LMICS and rename your sections Limits by Limits and perspectives

Line 849. Should be Third?

Lines 850-856. This is not a limitation because it was not the objective of your work to be able to generalize to Ghana and to all LMICS.

Lines 860-863. This sections could be merge with the first one to be more concise

Line 880. As stated before, I fail to see how this work has bridged the gap between policy and practice, and what you really mean with this statement. Which gap you are talking about? What is policy for you?. You have explored facilitators and barriers to collaboration at operational level, and address the importance of institutionalisation (closely linked to policy), but I don’t really see where you have improved the alignment between policy decisions and field practice.
