# Supplementary material for: Beyond silos: Drivers and barriers to intersectoral collaboration in zoonotic disease surveillance and response in the Greater Accra Metropolitan Area, Ghana
Source: PLoS One. 2026 Apr 16;21(4):e0347471. doi: 10.1371/journal.pone.0347471 (PMC13086352; doi:10.1371/journal.pone.0347471)
Supplement: Attachment — Submitted filename: Responses to Reviewers V2.docx [file pone.0347471.s005.docx]

**Responses to Reviewer’s Comments (Round Two)**

**Reviewer 1 Comments (Dr. Marion Bordier)**

We are grateful for your continued engagement with our manuscript and for your constructive feedback that has further strengthened our work.

For clarity, we have retained all comments and responses from Round 1. Our responses to your Round 2 comments follow immediately after the Round 1 responses for each point and are clearly labelled as 'Round 2'. Line numbers referenced in Round 2 responses are highlighted in yellow in the revised manuscript for easy identification.

**Abstract**

**1.** ***Comment (Round 1)****- Line 35: when you say “governance and surveillance organization”, is organization applied to surveillance and governance or to surveillance only? In both cases, it sounds a bit strange to me. I wonder if “surveillance governance and organization” would*

*not be more suitable.*

**Response (Round 1)** – Thank you for this insightful observation. We agree with your suggestion and have revised the phrasing to ‘surveillance governance and organization systems’ in the manuscript and subsequent figures. (Line 35)

***Follow-up Comment (Round 2)*** *- Sorry, but it is still not clear to me. I suggested “the governance and organization of surveillance”. What do you mean by organization systems? system of what? In addition, you announce factors influencing disease surveillance and response, but mention only surveillance here.*

**Response (Round 2) -** Thank you for this clarification. We agree that the previous phrasing remained ambiguous. We have now revised the text to: “the governance and organization of surveillance and response systems.” This wording directly reflects the scope of the study and clearly specifies what is being governed and organized. The change has been applied in the Abstract and throughout the manuscript where relevant (Lines 35–36, 380).

**2.** ***Comment (Round 1)*** *- Line 45: we usually prefer to use collaboration without ‘s’*

**Response** – Thank you, the ‘s’ has been removed. (Line 44)

***Follow-up Comment (Round 2)*** *- But it still remains in other parts of the manuscript.*

**Response (Round 2) -** Thank you for this clarification. I have reviewed the entire manuscript again to ensure consistent and intentional use of ‘collaboration’ versus ‘collaborations.’ In three sentences, I retained the plural form because the sentence refers to multiple discrete collaborative events involving different actors, which aligns with how participants described their experiences (Lines 37, 250 and 509). In all other remaining places, the term refers to collaboration as a general process, in which case the singular form is more appropriate.

These adjustments ensure clarity and accuracy while reflecting the intended meaning in each context.

**Introduction**

**3. *Comment (Round 1*** *- Line 57: I do not see how limited public health infrastructure can favour spill over. Can you clarify?*

**Response (Round 1) -** Thank you for this important clarification. You are correct that limited public health infrastructure does not directly favour spill over events. We have revised the sentence to clarify that while frequent human-animal interactions and weak surveillance systems create conditions for spill over, limited public health infrastructure facilitates the spread and impedes effective response. (Line 56-57)

***Follow-up Comment (Round 2)*** *- Still not clear to me. How weak surveillance systems creates conditions for spill over? The limited public health infrastructure facilitates the spread of what? I would move the weakness of surveillance to an additional factor that facilitate that impeded effective response.*

**Response (Round 2) -** Thank you for this important clarification. We agree with your point and have revised the sentence to clearly distinguish between factors that facilitate spill over events versus those that impede detection and response. We have now grouped weak surveillance systems together with limited public health infrastructure as factors that impede effective detection and response, rather than as conditions that create spill over.

The revised sentence now reads: “The risk of zoonotic disease transmission is particularly high in low- and middle-income countries (LMICs), where frequent human-animal interactions facilitate zoonotic spill over, while weak surveillance systems and limited public health infrastructure impede effective detection and response.” (Line 53-56)

This revision clarifies that frequent human-animal interactions are the primary driver of spill over events, while surveillance and infrastructure weaknesses affect our capacity to detect and respond to these events after they occur.

**4. *Comment -*** *Lines 75-77 soften the statement of lines 69-71 and should be put side-by-side.*

*Not sure that lines 71-74 are bringing relevant information in the context of this study.*

**Response -** Thank you for this helpful suggestion. We agree and have reorganized this section to improve logical flow. We have placed the two related statements about gaps in Ghana-specific research side-by-side, using the softer framing throughout. We have also removed lines 71-74 as you suggested, as they were not directly relevant to establishing the rationale for this study.

The revised paragraph now reads: “While research in other LMICs highlight the importance of institutional coordination and stakeholder engagement [13,15], empirical studies specifically examining intersectoral collaboration in Ghana's zoonotic disease surveillance and response (ZDSR) system are still emerging. Although some studies explore high-level One Health policy coordination in Ghana [16], little is known about how intersectoral collaboration occurs at the operational level within ZDSR systems” (Lines 68-73)

**5*. Comment*** *Lines 77-78. Why is it specifically crucial to understand collaboration dynamics when there is a high zoonotic disease burden and under-resourced health systems? The causal link is not obvious to me.*

**Response -** Thank you for pointing out that the causal link was not clearly articulated. We have revised this section to explicitly explain why understanding collaboration is particularly crucial in resource-constrained, high-burden settings. The revised text now clarifies that effective intersectoral collaboration is essential to maximize limited resources and prevent duplication of efforts in such contexts.

The revised text now reads: “Given Ghana's high zoonotic disease burden and under-resourced health systems [17–20], effective intersectoral collaboration is essential to make the most efficient use of limited resources and prevent duplication of efforts, making it critical to understand what facilitates or hinders collaboration in practice.” (Lines 73-76)

**6. *Comment:*** *Lines 84-90 sound more like a conclusion than an introduction to the study that will be presented after. Should be rephrased.*

**Response -** Thank you for this observation. We agree and have revised this paragraph to use more appropriate framing that introduces the study's aims rather than presenting findings.

The revised text now reads: “By examining the realities of operational-level intersectoral collaboration, this study aims to provide, evidence-based insights for developing training programs, policy adjustments, and collaboration strategies that are both effective and sustainable. Without this empirical foundation, policy initiatives alone may not translate into meaningful improvements in practice [21,22].” (Lines 80-84)

**Methods**

7*.* ***Comment -*** *Line 100: what do you mean by “which accounts for the higher participant numbers in one district”?*

**Response -** Thank you for this feedback. We agree that this information was presented prematurely at this point in the manuscript, before readers would have context about participant distribution. We have removed this phrase (from lines 91 - 92) and retained the explanation in the participant selection section (lines 159-161) where participant numbers by district are presented. Please see our response to Comment 12 for the revised explanation of participant distribution. The revised text on lines 91 – 92 now reads: “The broader project comprised multiple objectives including: (1) a rabies sectoral surveillance evaluation conducted in select districts in each area”.

8. ***Comment -*** *Line 103: what do you mean by component? Before, you are talking about objectives.*

**Response -** Thank you for catching this inconsistency in terminology. We have revised the sentence to clarify that this paper addresses one of those objectives.

The revised text now reads: “This paper uses the interview dataset from objective 2 of the study to identify specific factors influencing collaboration dynamics in the Greater Accra Metropolitan Area (GAMA).” (Line 94-97)

9. ***Comment -*** *Lines 139-140. They agreed, but were they ultimately interviewed?*

**Response -** Thank you for seeking this clarification. Yes, all three wildlife health participants who agreed to participate were ultimately interviewed. We initially did not include this detail at lines 136-137 because the final sample composition, including all three wildlife health participants and their specific roles, is presented in detail in lines 150-152. However, to provide complete transparency about the recruitment outcome and maintain parallel structure with the reporting of human and animal health sectors in this paragraph, we have added this information. The revised sentence now reads: “All three wildlife health participants contacted agreed to participate and were interviewed.” (Lines 136-137)

***10. Comment (Round 1****- We are also missing the distribution of participants across the 16 districts to assess the level of representativity of each district.*

**Response (Round 1) -** Thank you for this suggestion. We understand the importance of demonstrating representativeness. However, providing district-level distribution data would significantly compromise participant confidentiality in our study context. The animal health sector has very small numbers of practitioners at the district level - in many cases only 1-2 per district - and since we interviewed complete teams in most districts, a table showing participant counts would essentially create a map that readers familiar with the animal health sector could use to identify specific districts. For example, if a table showed "District X has 4 animal health

participants," colleagues in the sector would immediately know which district this refers to, as very few districts have 4 animal health officers. Combined with contextual details in quotes, this would compromise the anonymity we promised participants. We provide narrative information confirming that participants were recruited from across all 16 districts, with variation in numbers reflecting differences in sector sizes, purposive sampling of key ZDSR stakeholders, and one district's involvement in another study component (Lines 157-164). We believe this approach balances the need to demonstrate broad geographic representation with our ethical obligation to protect participant confidentiality in a small, highly interconnected professional community.

***Follow-up Comment (Round 2)*** *- Sorry, but it is not clear to me how you made your sampling. On one hand you are talking about purposive and convenience sampling, and then you say that you interviewed complete teams in most districts. In addition, you talk about 19 districts first, and then you say that participants were distributed across all 16 districts. Be more precise about the sampling strategy used at each stage (selection of district, selection of participants).*

**Response (Round 2) –** Thank you for this important feedback. We have substantially revised the participant selection section to provide greater clarity about our sampling strategy at each stage and to explain the progression from 21 eligible districts to the final 16 districts where interviews were conducted.

**Clarification of sampling approach:** We have reorganized the Methods section, moving detailed information about district selection from the Study Setting section into the Study design and participant selection section (Lines 117 – 119) where it is more appropriately positioned. We have clarified how purposive and convenience sampling were integrated. Districts and district heads were purposively selected based on their authority and direct involvement in ZDSR. However, convenience sampling elements allowed district authorities to choose whether to participate directly, nominate subordinates, or involve additional team members, provided all participants had direct ZDSR responsibilities (Lines 120 – 124).

**Clarification of "complete teams":** We have explained that for animal health, district teams were small (typically 1-4 officers per district), and in most cases all available animal health officers were interviewed, providing near-complete sectoral representation. For human health sectors, which have larger teams, key personnel with direct ZDSR roles were purposively selected (Lines 154 – 159).

**District progression (21→19→16):** We have added a detailed explanation of how the number of districts changed through the recruitment process:

- **21** districts qualified (had both human and animal health officers)
- Invitations were sent to all 21 districts, but due to incomplete or incorrect contact information, invitations reached 20 human health and 17 animal health district heads (one animal health officer oversaw two districts)
- Confirmations were received from 17 human health and 17 animal health district heads, covering **19** districts total
- Ultimately, interviews were conducted in **16** districts (human health representation in 13 districts, animal health representation in 10 districts, with only seven districts having both sectors represented)
- The reduction occurred due to data saturation, scheduling conflicts, participant availability, and time constraints (Lines 126 - 136)

These revisions provide a complete and transparent account of our sampling procedures at each stage of the study.

***11.*** ***Comment -*** *Lines 156-157 should be placed at the end of the previous paragraph*

**Response –** Thank you for the comment. We have placed that statement at the end of the previous paragraph. (now lines 152-153)

**12.** ***Comment -*** *Lines 158-160: I do not understand the causal link in this sentence*

**Response -** Thank you for requesting clarification of the causal link. We have revised this section to explicitly explain why one district contributed more participants. The revised text now clarifies that this district was selected for the rabies surveillance evaluation component (mentioned earlier in lines 91-92), which required visiting all health facilities and therefore enabled broader sampling for this study while maintaining data collection efficiency.

The revised text now reads: “One district’s human health sector, however, contributed a larger number of participants (n=16) due to its involvement in the rabies surveillance evaluation of the broader research project. This evaluation required visiting all public health facilities in that district, which enabled broader sampling of ZDSR personnel beyond the typical 1-4 key stakeholders interviewed in other districts.” (Lines 159-163). This revision makes the methodological rationale clear while maintaining transparency about sampling procedures.

13. ***Comment -*** *I am not sure we need lines 160-164*

**Response –** Thank you for the comment. We agree that this information is redundant, as differences in sector sizes and the purposive sampling approach are already explained earlier in the same paragraph. We have therefore removed this sentence to avoid repetition.

**14.** ***Comment -*** *Line 200: I would add discourse after participants. What do you think?*

**Response -** Thank you for this suggestion. We agree that adding ‘discourse’ clarifies that themes emerged from participants' interview narratives. We have revised the sentence to read: ‘All themes reported in this manuscript were derived from multiple participants’ discourse; no single theme was based on one individual’s perspective. (Lines 199-200)

**15.** ***Comment -*** *Lines 222-223 – why do you have selected no verbatims for one district?*

**Response -** Thank you for seeking this clarification. Those sentences were in response to your Round 1 concern about ensuring themes were not based on a few individuals. We have now made this clearer by providing details about the representativeness of illustrative quotes across districts.

To avoid overrepresentation, human health quotes from the district with highest participation (n=16) were excluded from illustrative examples, though animal health quotes from this district were retained for illustration. Additionally, all illustrative quotes presented in this manuscript represent 15 of the 16 districts. This is because one different district had only one sector interviewed and their verbatims were not optimal for illustrating the themes. All data from both situations were fully included in the analysis.

The revised text now reads: “Finally, to avoid overrepresentation, human health quotes from the district with the highest participation (n=16) were excluded from illustrative examples presented in this manuscript (though included in the analysis), while animal health quotes from this district were retained. Overall, the illustrative quotes presented in the manuscript represent participants from 15 of the 16 districts and across all organisational levels. One district is not represented in the quotes because only one sector was interviewed in that district and their verbatims were not optimal for illustrating the themes identified.” (Lines 220 – 227)

**Results**

**16.** ***Comment -*** *I find the text difficult to read because of all the quotes that interrupt the text. May be, you can embed them in the text. Example: Personal rapport between actors was one of the strongest enablers of collaboration. Friendly relationships fostered trust, encouraged communication, and facilitated informal information exchange. As one animal health informant explained: “It’s because we have the rapport...” (AH)*

**Response –** Thank you for this suggestion. We have revised the manuscript to integrate shorter quotes (one to two sentences) directly into the text to improve readability, as you demonstrated in your example. However, we have retained separate formatting for a few longer quotes (three or more sentences) to maintain clarity and prevent overly dense paragraphs. This approach balances narrative flow with the need to present substantial participant narratives clearly, which is consistent with standard qualitative research reporting practices.

**17.** ***Comment -*** *I would also suggest not to put one verbatim after each statement, but to articulate some statements together and choose the best illustrative verbatim (and the easier one to understand for people not familiar with the context, because a lot of verbatims are difficult to understand)*

**Response –**Thank you for this valuable feedback. We have substantially revised the Results section to address these concerns. We have reduced the total number of verbatims throughout the manuscript, removing quotes that were redundant with surrounding prose or that did not add substantive illustration. We have also restructured the text to articulate multiple related statements together before providing the most illustrative quote, rather than including a quote after each individual statement. Additionally, we have shortened longer quotes to focus on key phrases and added contextual clarifications in brackets where necessary to improve comprehension for readers unfamiliar with the Ghanaian context. These revisions significantly improve the readability and clarity of the Results section while maintaining the rich qualitative evidence that supports our findings.

***18.*** ***Comment -*** *It would be helpful to define what an assembly coordinator is as they are mentioned in a lot of verbatims*

**Response –** Thank you for this suggestion. We have added a definition of assembly coordinator in the text (Lines 257 - 261).

**19.** ***Comment -*** *Line 237-238. What is the difference between quotes integrated in the text and the ones provided in the supplementary file 1?*

**Response -** Thank you for seeking this clarification. The quotes integrated in the manuscript text have been lightly edited for clarity and readability - this includes minor paraphrasing, addition of contextual clarifications in brackets, and shortening where appropriate to maintain focus on key points. The supplementary file (S1) provides two types of content: (1) the complete, unedited verbatims for quotes that appear in the manuscript, allowing readers to see the full context and original wording, and (2) additional illustrative quotes that support the themes but were not included in the manuscript due to space constraints and the previous request to reduce the number of quotes. This approach balances readability in the main text with transparency and completeness in the supplementary materials.

***20.*** ***Comment -*** *line 246: you need to better explain this quote. What is “it” referring to?*

**Response -** Thank you for pointing out this ambiguity. In response to your earlier suggestion to reduce verbatims and improve clarity, we have removed this quote as it was redundant with the surrounding text and did not add substantive illustration beyond what was already stated in the prose.

**21.** ***Comment -*** *Lines 301-303 – It is not clear to me what you want to stress out. The professional recognition of who by whom create motivation of who?*

**Author Response –** Thank you for seeking clarification. In response to your suggestion to reduce verbatims and improve readability, we have removed this quote but have clarified the meaning in the prose. The revised text now states: “In addition to these practical benefits, professional recognition was also a motivator, as some participants noting that collaborative activities enhanced the professional standing or image of the officer, their unit or institution” (Lines 295 - 297). This clarifies that collaborative activities provided recognition that motivated individual officers to engage in intersectoral work.

***22.*** ***Comment -*** *Lines 304: Self-protection is illustrated later lines 312-314. So why mentioning it here?*

**Response –** Thank you for catching this organizational issue. You are correct that self-protection was mentioned prematurely before being illustrated. We have restructured this section to separate compliance motivations (proactive) from self-protection motivations (reactive), ensuring each theme is introduced only when supported by illustrative quotes (Lines 298-309).

**23.** ***Comment -*** *Lines 310. What is NADMO? If we do not know, we do not see the collaboration here.*

**Response –** Thank you for this important clarification. We have now defined NADMO (National Disaster Management Organization) and explained their role in the collaboration. The revised text clarifies that animal health officers were legally required to obtain NADMO signatures for avian influenza carcass destruction documentation, making this a mandated collaborative touchpoint (Lines 300-304).

***24.*** ***Comment -*** *Lines 341-345. I do not see how this verbatim illustrate alignment between priorities of actors. The AH actor is talking about views, not priorities. This is more in line with the fact they are both new? Cf lines 251-256*

**Response -** Thank you for this important observation. You are correct that this quote does not clearly illustrate alignment of ZDSR-specific work priorities without additional context from earlier in the interview. While the animal health participant was describing shared vision and goals with the agriculture director (in contrast to their failed relationship with human health), this connection requires too much backstory to be clear in the text. We have removed this quote to ensure each quote clearly and directly illustrates the specific theme under discussion. The revised section now focuses on examples that more directly demonstrate alignment or misalignment of ZDSR priorities (Lines 322-338).

***25.*** ***Comment -*** *Lines 556-557. Lack of exposure to what? We need to better see the link with the theme “knowledge and training”.*

**Response -** Thank you for requesting this clarification. We have revised the text to explicitly explain that "lack of exposure" refers to the absence of training and education about other sectors' roles and responsibilities in ZDSR. The revised text now clearly connects this educational gap to the knowledge and training theme by stating: “Sectoral silos persisted due to limited training and education about other sectors' roles and responsibilities in ZDSR. This lack of educational exposure to the work and relevance of other sectors meant that collaboration with certain sectors was simply unimaginable to some participants” (Lines 462 - 465). This revision makes the connection between training gaps and collaboration barriers more explicit.

***26. Comment (Round 1) -*** *Why did you address motivation, expectations, and perceived benefits separately from factors driving and hindering collaboration?*

*To me they are also factors that influence collaboration. And you present them as such at the*

*beginning of the discussion (lines 621-622).*

*Please clarify and explain how everything is linked, or introduce them as specific themes in*

*part A. and merge everything together.*

**Response (Round 1) -**

Thank you for this valuable comment. We agree that motivations, expectations, and

perceived benefits all influence collaboration and should be integrated rather than treated

separately. We have thus revised the Results section to reflect this (Lines 226 - 683).

Specifically, we have:

- Reorganized our results into ‘Factors Influencing Intersectoral Collaboration in ZDSR’ with two categories: Individual factors (relationships, personal initiative, motivations, perceptions of superiority, shared goals), and Structural factors (governance, visibility, workforce availability, knowledge, and resource constraints). Positive collaborative outcomes fall within both individual and structural factors.
- Integrated **‘**motivations’ into the ‘individual factors’, emphasizing that they represent participants' underlying incentives for engaging in collaboration.
- Renamed ‘perceived benefits’ to ‘Positive collaborative outcomes’ and placed it as a factor that falls under both individual and structural factors.
- Separated ‘general expectations for future collaboration’ as stakeholder aspirations to clearly distinguish aspirational views from demonstrated influences on current collaboration.

This restructuring creates a more cohesive framework linking motivations, experiences, and expectations as interconnected influences on collaboration.

***Follow-up Comment (Round 2 -)*** *Ok, but you should mention in the introduction of the Results’ section the positive outcomes, the facts that they may be Individual or Structural factors and that’s why you address them separately.*

**Response (Round 2) -** Thank you for this helpful suggestion. We have added an explanation in the Results introduction about positive collaborative outcomes and why they are addressed separately. The revised text now states: ‘A third category, Positive Collaborative Outcomes, is addressed separately as these factors are consequential, arising from successful collaboration and operating across both the individual and structural domains to create reinforcing cycles.’ (Lines 234 -237)

**Discussion**

***27. Comment -*** *There is still work to be done on the discussion. It could be more concise and better articulated. In addition, there are often very broad and somewhat vague recommendations, and we would like the authors to go a little further in explaining how these recommendations could be implemented in the context of Ghana and in light of their work.*

**Response -** Thank you for this important feedback. We have substantially revised the Discussion section to make it more concise and provide concrete, context-specific recommendations. We have reduced the overall length by approximately 20% by consolidating overlapping sections and eliminating redundancy. More importantly, we have replaced broad recommendations with specific, implementable mechanisms grounded in Ghana's context in a dedicated section called ‘Structural Solutions – Optimizing institutional architecture for One Health’ (Lines 637-695). These include specific reforms to existing governance structures, mandatory protocols, educational reforms, and data-sharing mechanisms - each directly linked to barriers identified in our findings. Please see our detailed response to Comment 31 for a comprehensive explanation of how these recommendations address concerns about institutionalization creating new barriers or reinforcing existing inequities.

***28****.* ***Comment -*** *Line 723. What do you mean by inequities? Is that linked to power imbalances that you are mentioning below? If yes, the link between the two paragraphs should be clearer.*

*I can see that you are also mentioning inequities line 732.*

**Response -** Thank you for requesting this clarification. Yes, inequities and power imbalances are linked concepts. In response to requests for conciseness, we have restructured and condensed this section into three distinct but related structural challenges:

**Inequitable access to collaborative opportunities** - where participation depends on networks and assertiveness rather than expertise, systematically excluding those without strong connections. (Lines 588 - 592)

**Fragility of informal structures** - vulnerability to staff turnover due to lack of institutional memory. (Lines 593 - 596)

**Perceived professional hierarchies** - subjective perceptions of sector dominance that arise when formal role definitions are absent. (Lines 597-601)

The first point addresses inequitable access, while the third addresses the power imbalance concerns. These are related - systems requiring constant self-advocacy (point 1) can reinforce perceived hierarchies (point 3), as those with better networks and assertiveness (often human health) gain dominant positions. Both stem from weak institutional structures and require structural solutions, though they operate through different mechanisms.

***29****.* ***Comment -*** *Lines 724. I would take the opportunity of this section to discuss the fact that participants perceived that medic trained in public health are more collaborative than the ones who are not.*

**Response** - Thank you for this excellent suggestion. We have added discussion of this finding in the recommendations section (Structural Solutions – Optimizing institutional architecture for One Health) to illustrate potential pathways for addressing perceived hierarchies. The revised text now highlights how public health training was associated with more collaborative attitudes and respect for other sectors' expertise, suggesting that institutionalizing One Health education could help neutralize perceived hierarchies by establishing shared understanding of sectoral interdependence (Lines 666 - 674). This connects our finding about training to the broader discussion of how formal structures and education can reduce reliance on personal networks and subjective status perceptions.

**30.** ***Comment -*** *Line 726. I understand your point but, to me, power imbalance is real as the human health sector is usually more well-resourced than the animal health sector.*

**Author Response** - Thank you for this important clarification. We agree that the human health sector is typically better resourced than the animal health sector. We have revised the text to explicitly acknowledge these objective disparities while clarifying our analytical focus. The revised text now states: “Participants reported perceived human health dominance likely stemming from visible disparities in workforce capacity and resources, given that the human health sector is typically better resourced. Without clearly defined cross-sectoral roles, actors’ default to interpreting hierarchies subjectively, creating barriers even where objective power differences are minimal” (Lines 597–601). This revision acknowledges real structural inequalities while retaining our point that unclear role definitions can amplify perceived hierarchies beyond material resource gaps.

**31.** ***Comment -*** *Line 737-740. But what do you propose for this to happen? We have seen that almost all African countries have now One Health Platforms, but they are barely non-functional and inequities as well as power imbalances persist. So how can we make OH institutionalisation working? Some studies show that too rigid institutional mechanisms also hamper collaboration because people see a risk to lose their autonomy and to be “forced” to do things they do not want to do (such as sharing data).*

*In addition, I am not sure that institutionalization is synonymous of equity. We have seen that institutionalization may also reinforce inequity. For instance, all international regulations which are mainly focusing on priorities of the “North” shape how funding is allocated to countries and to specific national counterparts. However, those countries might have other priorities than the ones the international standards are focusing on, and the funding mechanisms may create competition between national actors to accede fundings*

**Response** - This comment relates to our response to Comment 27 regarding concrete recommendations. Thank you for this excellent point that our original framing inadequately addressed. It is correct that rigid institutionalization can create new barriers and that formal structures do not automatically ensure equity; indeed, they may reinforce existing power imbalances.

We have revised our recommendations section to explicitly acknowledge this tension (Lines 637 - 695). Our approach differs from typical "create new OH platforms" recommendations in three ways:

First, we propose adapting existing structures (PHEMCs) rather than creating parallel One Health platforms that often remain non-functional. This avoids the pitfalls of new bureaucratic layers while leveraging established governance mechanisms.

Second, we acknowledge that institutionalization alone is insufficient. Our recommendations emphasize not just formal inclusion but mechanisms to ensure meaningful participation: explicit membership mandates (to prevent ambiguous representation), routine interaction through scheduled meetings (to build familiarity and trust), and communication protocols that maintain operational autonomy while enabling coordination (lines 643-681). This addresses the concern about rigid mechanisms that threaten perceived autonomy.

Third, regarding equity: we explicitly state that "complete resource equity is unlikely where human health receives greater political attention" (Lines 639-642), acknowledging structural power imbalances. Rather than claiming institutionalization creates equity, we argue that modest structural adjustments can mitigate inequities, for example, formal PHEMC membership prevents animal health exclusion due to administrative ambiguity, even if resource disparities persist. We also acknowledge a critical limitation: our district-level recommendations cannot address wildlife health marginalization, which requires national workforce investment beyond local governance reforms (lines 692-695).

The reviewer's point about international funding mechanisms creating competition among national actors and potentially prioritizing 'Northern' agendas is well-taken and represents an important structural dimension. However, our study focused specifically on domestic operational-level governance barriers, and our data do not speak directly to how international funding shapes collaboration dynamics. We have added a note in our limitations section acknowledging this as an important area for future research (lines 713-717), as understanding how international frameworks interact with domestic collaboration dynamics is crucial for comprehensive One Health implementation.

**32.** ***Comment -*** *Line 750. Poor enforcement of what?*

**Response** - Thank you. We have clarified this vague phrasing; and instead describe the specific implementation failures identified in our findings: (1) collaboration occurring only during outbreaks rather than routinely, and (2) district authorities failing to act on submitted collaborative plans (illustrated by the rabies control proposal that was requested but never implemented). The revised text (Lines 604-607) now specifies: “Event-driven rather than routine collaboration and lack of follow-through on submitted plans (such as rabies control proposals) disrupt continuity and limit the institutionalization of collaboration.” This clarifies that the problem is not enforcement of existing mandates per se, but rather lack of political will and administrative follow-through at district level.

***33.*** ***Comment -*** *Line 754-756. Ok, but how to achieve this? In the previous paragraph, you are calling for more institutionalisation, but now you say that it is not enough for collaboration to be effective.*

*You need to discuss these two points together and propose concrete solutions to that or at least way of improvement.*

**Response** - The reviewer identified an important contradiction in our original framing: we called for institutionalization while simultaneously suggesting it was insufficient. We have resolved this by:

1. Removing the contradictory sentence from the Discussion section. The original text created confusion by stating institutionalization was needed, then immediately undermining that claim.
2. Relocating the discussion of "how to achieve effective institutionalization" to our new Recommendations section (lines 637-695), where we address it substantively rather than as an afterthought. Our recommendations distinguish between:

Policy adoption (establishing mandates, creating structures)

Operational implementation (ensuring compliance, accountability, sustained function)

We argue that both are necessary: Ghana needs formal mandates for animal/wildlife health inclusion (policy level) AND mechanisms ensuring these mandates translate into practice (implementation level). For example, IDSR guidelines establishing PHEMCs represent policy adoption, but ambiguous wording ("District Director of Veterinary/Agricultural Services") allows de facto exclusion, an implementation gap requiring operational clarification.

3. Explicitly acknowledging (per Comment 31) that institutionalization can reinforce inequities if not carefully designed.

Our recommendations therefore emphasize not just formal structures but how those structures function: routine meetings build familiarity, clear membership prevents exclusion, communication protocols preserve autonomy while enabling coordination. This responds directly to the concern that rigid institutionalization may be counterproductive.

***34.*** ***Comment -*** *Line 769. I do not understand what this paragraph is adding to what is described previously. You mention physical proximity but then you are mainly referring to visibility and are redundant with the previous paragraph. These two paragraphs should be merged and ideas better articulated.*

**Response** - We agree that the previous draft contained redundancies and that the concepts of visibility, inaccessibility and proximity were not sufficiently distinguished. We have merged these paragraphs into a single, cohesive argument that provides a clearer logical progression. The revised section can be found at lines 608 – 617.

To support this distinction, we have added a specific finding to the Results section (Lines 437-440) describing instances where human and animal health sectors were located in the same building but had no history of collaboration due to a lack of formal mandates. This clarifies that visibility, accessibility and proximity are prerequisites but not the final drivers of engagement.

***35.*** ***Comment -*** *Lines 825-827. To my knowledge, it has been already addressed in the literature. See for instance:* [*https://doi.org/10.1186/s12889-022-13878-3*](https://doi.org/10.1186/s12889-022-13878-3)*.*

**Response** - We thank the reviewer for this observation and the suggested reference (Nana et al., 2022). While we agree that systemic barriers are documented regionally, we have clarified that a significant gap exists regarding Ghana’s specific operational-level architecture. We have updated the text (Lines 706-708) to ground our work in existing West African scholarship while highlighting our specific contribution to the Ghanaian context.

***36.*** ***Comment -*** *Lines 829-833. I don't see how you are proposing a roadmap; at best, you are proposing avenues for reflection and points to consider.*

**Response** - We agree that "roadmap" was an overstatement. We have replaced this term with "evidence-based insights" to better reflect the nature of our findings and based on comment 37 we have moved this section to the conclusions section (Lines 732-734).

***37. Comment -*** *Lines 824- 833. I do not see how this paragraph relates to Bridging Expectation–Reality Gaps. It looks more like a summary of what you have highlighted before. It could be merged with your paragraph about the potential generalisation of your results to Ghana and LMICS and rename your sections Limits by Limits and perspectives.*

**Response** - We agree with the reviewer’s assessment of the section's flow. Following your suggestion, we have renamed the section “**Limitations and Perspectives**” (Lines 697-717) and merged the summary paragraph here and in the conclusions section (Lines 732-734). This restructuring allows for a better connection between the study's limitations and the broader implications for Ghana and other LMICs.

***38. Comment -*** *Line 849. Should be Third?*

**Response** - Thank you, we have corrected this. (Line 705)

**39.** ***Comment -*** *Lines 850-856. This is not a limitation because it was not the objective of your work to be able to generalize to Ghana and to all LMICS.*

**Response** - We agree with the reviewer that statistical generalizability was not the objective of this qualitative study. However, to ensure transparency regarding the study’s scope (as discussed in the previous round of review), we have retained this section but reframed it. We have shifted the focus from ‘lack of generalizability’ to ‘contextual specificity,’ emphasizing that our study prioritized depth within an urban metropolitan context. We have also clarified that while the findings are transferable to similar LMIC settings, their manifestations may vary in different geographical contexts. (Line 705- 712)

***40.*** ***Comment -*** *Lines 860-863. These sections could be merge with the first one to be more concise.*

**Response** - We agree with the reviewer’s suggestion for greater conciseness. We have merged the limitation regarding 'direct impact measurement' into the first paragraph concerning 'self-reported data.' This consolidates our discussion of data validity and results in a more streamlined presentation of the study's constraints. (Lines 698 - 701)

***41.*** ***Comment -*** *Line 880. As stated before, I fail to see how this work has bridged the gap between policy and practice, and what you really mean with this statement. Which gap you are talking about? What is policy for you? You have explored facilitators and barriers to collaboration at operational level and address the importance of institutionalisation (closely linked to policy), but I don’t really see where you have improved the alignment between policy decisions and field practice.*

**Response** - We appreciate the reviewer's important clarification and agree that the original phrasing was imprecise and overstated our contribution. We have substantially revised the conclusion to reflect more accurately what our study demonstrates.

**What we changed:**

We removed the claim that our study “bridged the gap between policy and practice.” The reviewer is correct, our study did not improve alignment between policy decisions and field practice, nor did we conduct comparative policy analysis.

**What we did:**

Our study documents the operational realities that explain why One Health policies fail to materialize in practice at Ghana's district level. Specifically, we provide empirical evidence showing:

- How collaboration currently functions through informal, fragile personal networks rather than formal institutional structures.
- Why participants engage for pragmatic rather than idealistic reasons.
- What specific structural barriers (ambiguous committee membership guidelines, absent introduction protocols, inadequate workforce) prevent policy intentions from being realized

**Our revised framing:**

We now position our contribution as providing operational-level insights essential for translating policy aspirations into functional practice (Lines 719-736). Rather than claiming to have bridged the gap, we document where and why the gap exists, and what systemic reforms are needed to address it. This is more accurate and better reflects our study's methodology and findings.

We thank the reviewer for pushing us to be more precise about our contribution.

**Reviewer 2 Comments**

Thank you very much for reviewing our work and for your feedback. We have tried our best to address your suggestions.

1. ***Comment (Round 1) -*** *I consider this manuscript to be of interest to machine learning researchers in biological systems as well as readers of this journal. As such, I am generally supportive of publication with a few minor comments. While the authors focus on reflexive thematic analysis, there have been recent studies using advanced machine learning methods, such as t-SNE method, which can be used efficiently for classification as well as detecting trends in biochemical systems, which should be noted: Environ. Sci. Technol. Lett. 2023, 10, 1017–1022 and Mar. Genom. 2020, 51, 100723. In particular, these prior studies showed that t-SNE can give classification of results that could help diagnose trends in biochemical systems. I am not asking the authors to carry out such t-SNE calculations, but these recent applications of t-SNE should also be noted.*

**Response (Round 1) -** We appreciate the reviewer's positive assessment and support for our manuscript. We're also grateful for their acknowledgement of the paper's potential interest to machine learning researchers. However, we'd like to respectfully clarify that our study focuses on health systems research, specifically exploring the qualitative aspects of intersectoral collaboration within zoonotic disease surveillance. While the cited machine learning methods are indeed powerful for analyzing pure biological or biochemical systems, our research deals with complex human interactions, governance, and social dynamics, which are distinct from the types of data typically analyzed by t-SNE for classification or trend detection in biochemical contexts. Our chosen methodology, reflexive thematic analysis, was specifically selected to deeply understand these nuanced human experiences, which are central to health systems.

***Comment (Round 2)*** *- The authors did not address my previous review comments. I have re-pasted them below. Until the authors address them, I recommend re-review of this manuscript.*

**Author Response (Round 2)** – We thank the reviewer for their comments. As stated in our previous response, this study is a qualitative health systems research project using reflexive thematic analysis. Our data consists of human perceptions and governance experiences, which are not high-dimensional biochemical datasets suitable for t-SNE analysis. We have maintained the focus of the manuscript on the social and structural dynamics of One Health, as we believe introducing machine learning methods for biochemical systems would fall outside the scope of the study and potentially mislead readers regarding our methodology.
