# Supplementary material for: Beyond silos: Drivers and barriers to intersectoral collaboration in zoonotic disease surveillance and response in the Greater Accra Metropolitan Area, Ghana
Source: PLoS One. 2026 Apr 16;21(4):e0347471. doi: 10.1371/journal.pone.0347471 (PMC13086352; doi:10.1371/journal.pone.0347471)
Supplement: Attachment — Submitted filename: Response to Editor .docx [file pone.0347471.s006.docx]

**Response to Editor – Additional Comments**

Dear Editor,

We sincerely thank you for your careful evaluation of our manuscript.

1. **Reviewer 2 citation requests**

We appreciate your guidance regarding Reviewer 2’s request to cite their previous work. As you noted, the suggested citations appear to fall outside the scope of the present study. In line with your recommendation, we have not incorporated these citations, as they are not directly relevant to the objectives or findings of this manuscript.

2. **Reporting of qualitative research (COREQ / EQUATOR guidelines)**

Thank you for highlighting the importance of comprehensive reporting for qualitative and mixed-methods research.

In response:

- We have completed and submitted the COREQ checklist.

- We confirm that all key elements recommended for qualitative reporting—including research objectives, sampling strategy, data collection procedures, analytical approach, reflexivity, and study limitations—are fully addressed within the manuscript and/or detailed in the COREQ checklist.

As such, no further changes to the manuscript were required, as the submitted version already complies with these reporting standards. The COREQ checklist serves to clarify and transparently indicate where this information is presented.

3. **Inclusivity in global research**

We appreciate the opportunity to address the journal’s policy on inclusivity in global research.

In response:

- We have carefully cross-checked and resubmitted the Inclusivity in Global Research questionnaire as a Supporting Information file to ensure that all referenced page numbers accurately correspond to the current version of the manuscript.

- The manuscript already includes the relevant ethical, cultural, and collaborative considerations, and references to this checklist are clearly indicated and/or further clarified where appropriate.

4. **Summary**

We are grateful for your guidance, which has helped ensure that our manuscript aligns fully with the journal’s reporting and policy requirements. We believe that the submitted materials now comprehensively address all points raised.

We thank you for your time and consideration and hope the manuscript is now suitable for publication in PLOS ONE.

Kind regards,

Joannishka Dsani, DVM, MA

PhD Candidate

Centre for Development Research,

University of Bonn, Germany
